# Participant ascertainment is differentially related to phenotypic characteristics and alcohol‐related genetic liability in a sample with severe alcohol use disorder

**DOI:** 10.1111/acer.70276

**Published:** 2026-03-09

**Authors:** Alexis C. Edwards, Kristin Passero, Michelle Eglovitch, Kathryn Polak, Anna Beth Parlier‐Ahmad, Enkelejda Ngjelina, Mallory Stephenson, Severine Lannoy, Dace Svikis, Kenneth Kendler

**Affiliations:** ^1^ Department of Psychiatry, Virginia Institute for Psychiatric and Behavioral Genetics Virginia Commonwealth University Richmond Virginia USA; ^2^ Department of Psychology Virginia Commonwealth University Richmond Virginia USA; ^3^ Department of Cellular, Molecular, and Genetic Medicine Virginia Commonwealth University Richmond Virginia USA

**Keywords:** alcohol use disorder, ascertainment, comorbidity, polygenic score

## Abstract

**Background:**

Alcohol use disorder (AUD) is a common substance use disorder associated with a range of sociodemographic, behavioral, and genetic factors. The current study characterizes variation in such factors as a function of ascertainment strategy in a sample of individuals with a lifetime history of severe AUD.

**Methods:**

Participants (*N* = 10,804) were recruited through substance use treatment facilities or through online outreach/advertisement in the United States and completed a survey that assessed a range of alcohol‐related variables, sociodemographics, psychopathology, and personality. Participants were asked to provide a saliva sample for DNA extraction and genotyping. Participants' survey responses and their polygenic risk for multiple alcohol outcomes were compared as a function of ascertainment strategy (“clinic” vs. “online”) using *t* and chi‐square tests.

**Results:**

Ascertainment strategy was significantly associated with many phenotypic variables, although effect sizes were generally small. In general, clinic participants reported more adverse outcomes, such as higher AUDIT‐C scores, longer duration of alcohol problems, antisocial behavior symptom counts, and four of five impulsivity facets. However, online participants reported more problems with depression. Polygenic risk scores differed by ascertainment strategy only for participants of European descent. Clinic participants' scores were higher for AUD, AUDIT‐C, drinks per week, and problematic alcohol use. The groups' scores did not significantly differ for typical maximum drinks in 24 h.

**Conclusions:**

Individuals with severe AUD exhibit heterogeneity across many risk domains, particularly for alcohol‐related measures. This heterogeneity can be captured through the ascertainment of study participants via diverse modalities, improving representativeness and potentially facilitating gene identification efforts.

## Graphical abstract

Using multiple recruitment modalities for studies of individuals with alcohol use disorder can expand the phenotypic and genetic heterogeneity of a sample, potentially making it more representative.

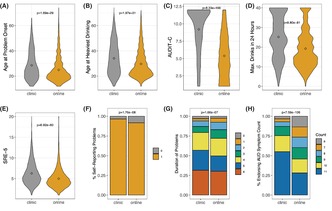

## INTRODUCTION

Alcohol use disorder (AUD) is an important public health concern related to morbidity and mortality worldwide (GBD 2016 Alcohol Collaborators, 2018; World Health Organization, 2018). Alcohol misuse and AUD have substantial personal, interpersonal, and societal consequences. In 2018, they contributed to hundreds of adverse health outcomes (e.g., cardiovascular and liver disease) as well as to other‐ and self‐directed violence (World Health Organization, 2018). In addition, alcohol use and AUD are among the costliest public health burdens, including within middle‐ and high‐income countries such as the United States (Rehm et al., 2009). In 2023, 28.9 million people in the United States aged 12 and older had AUD (SAMHSA, 2023). Importantly, worldwide per capita alcohol consumption is predicted to increase over the next decade, which carries a potential increase in disease burden (World Health Organization, 2024).

Risk for AUD is complex and multifactorial. For example, risk varies as a function of sociodemographic characteristics: Higher educational attainment was associated with a reduced frequency of high‐risk drinking in a US sample (Grant et al., 2017) and lower risk of AUD in a Swedish study (Calling et al., 2019). In addition, being married or partnered is also typically, though not always, associated with lower risk of alcohol problems (Hasin et al., 2007; Kendler et al., 2016). AUD is highly comorbid with other mental health conditions: 43.9% of individuals with a lifetime AUD had at least one other lifetime mental health disorder in a study of international data (Glantz et al., 2020). AUD risk was elevated among individuals with other drug use disorders, major depressive disorder, and personality disorders, among other conditions; these associations were typically stronger for those with severe AUD (Couvy‐Duchesne et al., 2018; Grant et al., 2015). Importantly, risk of AUD is also related to non‐clinical behavioral phenotypes. Features of personality and temperament—such as negative emotionality (e.g., neuroticism), positive emotionality (e.g., extraversion), and impulsivity in particular—have been implicated as indicators of or risk factors for AUD (Boschloo et al., 2013; Haeny et al., 2019; Ruiz et al., 2003; Slutske et al., 2002), though associations are not entirely consistent across studies (Mulder, 2002).

Biological factors must also be considered to fully understand the constellation of influences on AUD risk. AUD is moderately heritable, with twin and adoption studies yielding an estimate of 0.49 (Verhulst et al., 2015). Although variants in genes responsible for ethanol metabolism have been strongly implicated as risk/protective genetic factors (Edenberg, 2007), they explain little of the total genetic variance underlying AUD risk, which is highly polygenic. Recently, large‐scale analyses have been undertaken to characterize the genetic liability to AUD and other alcohol phenotypes (e.g., problematic alcohol use, maximum habitual alcohol use) (Deak et al., 2022; Kranzler et al., 2019; Liu et al., 2019; Sanchez‐Roige et al., 2019; Walters et al., 2018; Zhou et al., 2020). These studies have implicated variants beyond those within the alcohol dehydrogenase and aldehyde dehydrogenase genes, and have confirmed, using molecular genetic data, the extensive genetic correlations between alcohol‐related measures and a range of behavioral measures, including psychopathology.

Importantly, sample ascertainment plays a critical role in the potential success of genome‐wide association studies (GWAS) and has implications for the interpretation of findings. Efforts to identify genetic risk variants for major depression (MD) provide an example. While early GWAS of MD did not identify genome‐wide significant variants (Major Depressive Disorder Working Group of the Psychiatric GWAS Consortium et al., 2013), a subsequent study using a smaller sample of Han Chinese women with recurrent MD implicated two loci, which were replicated in an independent sample (Converge Consortium, 2015). Furthermore, the varying stringency of phenotype definition corresponds to differences in the underlying genetic architecture of MD. Cai et al. (2020) found that when MD cases were characterized using “minimal phenotyping”—that is, through a self‐report of having “seen a doctor for nerves, anxiety, tension or depression”—rather than through a more thorough assessment of symptoms, GWAS using the former approach disproportionately identified genetic variants that were not specific to MD. Thus, efforts to more narrowly dissect the genetic nature of AUD could benefit from a similar approach of analyzing only participants who are thoroughly assessed for Diagnostic and Statistical Manual (DSM) criteria and found to be severely affected.

In the current report, we describe the Genes, Addiction, and Personality (GAP) Study. The study was undertaken to bolster the effort to identify genetic variants implicated in AUD by recruiting participants with a lifetime history of severe AUD, operationalized by the endorsement of 6 or more DSM‐5 AUD criteria (American Psychiatric Association, 2013). We aimed to capture the heterogeneous nature of those AUD cases by recruiting participants through two modalities: at substance use treatment facilities and through online efforts, whom we refer to as “clinic” and “online” participants, respectively. The vast majority of individuals with AUD do not receive treatment: Only 7.8% of US adults with past‐year AUD received treatment during that year (SAMHSA, 2024), and approximately 20%–24% receive treatment during their lifetime (Venegas et al., 2021). It is therefore critical to include individuals from non‐clinical samples to improve the representativeness of findings. In this descriptive report, we characterize the sample with respect to sociodemographic features, alcohol and other substance use profiles, and relevant behavioral and psychological measures. We further investigate whether participants recruited through the two distinct ascertainment strategies (clinic vs. online) differ phenotypically. Finally, we present preliminary analyses comparing these two groups with respect to aggregate genetic liability for alcohol‐related outcomes.

## MATERIALS AND METHODS

The Genes, Addiction, and Personality (GAP) Study recruited participants for a GWAS of severe AUD. Ultimately, the sample will contribute to consortium efforts to identify genetic risk variants. Here, we describe findings using a data freeze of participants recruited December 2018 through December 2023. As noted below, data collection spanned the onset of the COVID pandemic, which began approximately in March 2020.

### Participant recruitment

Participants were recruited through two complementary approaches: online efforts and recruitment at substance use treatment facilities. Online participants were recruited through Faces and Voices of Recovery (FVR) and She Recovers, two online recovery communities; targeted advertisements on Facebook, a social media platform; and ResearchMatch (www.researchmatch.org), a national recruitment registry. Brief information blurbs and a link to the online survey were placed on the FVR and She Recovers websites. Online, participants were led to a REDcap (Harris et al., 2009, 2019) eligibility screener (see Table S1) directly from the targeted Facebook advertisements or links provided by ResearchMatch, etc. As part of the eligibility screener, online participants were asked whether they would be willing to provide a saliva sample for DNA analysis, and only those who answered affirmatively were administered the full survey.

At substance use treatment facilities, participants were recruited in‐person through use of posters and flyers, targeted presentations, and direct client approach. Clinic participants then consented to the survey component of the study, and if they met criteria for lifetime severe AUD (determined via an algorithm embedded in the survey software), they were asked to further consent to the DNA component of the study. Clinic participants who did not meet criteria for severe AUD still provided survey data, but they are not included in the current report. Further information on the methodology of in‐person and online recruitment, including exclusion and eligibility criteria, is in Table S1.

Participants provided written consent for the survey and for the provision of a saliva sample for DNA extraction. The protocol was approved by the Institutional Review Board at Virginia Commonwealth University (protocols HM20001578 and HM20013108).

### Phenotypic assessment

Participants were administered a survey designed to take approximately 15–20 min to complete if all sections were relevant. Sections could be skipped if they did not apply to the participant (e.g., those who had never smoked cigarettes were not administered the section on nicotine dependence). Full details of the assessments are provided in Table S2. Briefly, the survey covered the following topics: sociodemographic characteristics, alcohol use and problems, nicotine dependence, opioid use and problems, other illicit substance use, personality, impulsivity, adult antisocial behavior, depressive symptoms, resilience, and family history of alcohol problems. Note that the current report includes individuals who took part in our initial pilot study (*N* = 390, 3.6% of the total analytic sample; collected 2014–2015), which followed the same recruitment procedures as described above. However, some phenotypic assessments were not included in the pilot survey, which leads to varying missingness in the current report.

### Statistical analyses

We report the basic descriptive statistics (e.g., means, standard deviations, and distributions) for variables of interest. To assess whether participant characteristics differed as a function of ascertainment strategy (clinic vs. online), we conducted *t*‐tests for continuous variables and chi‐square tests for ordinal or categorical variables. Effect sizes were quantified using Cohen's *d*, Cramer's *V*, and a rank‐biserial estimate for the Wilcoxon test. We applied a Benjamini–Hochberg False Discovery Rate to adjust for multiple tests.

### Saliva collection, DNA extraction, and quality control

Participants provided 4 mL of saliva using Oragene OGR‐500 collection tubes from DNA Genotek. For clinic participants, study staff were available on‐site to confirm that an adequate sample was provided (e.g., sufficient volume and no food contamination). If online participants returned samples that were of low quality, a second sample was requested. Saliva samples were transferred to the molecular laboratory at Virginia Commonwealth University for DNA extraction. Table S3 provides details on the sample size for genetic analyses in the current report.

DNA samples meeting internal quality control standards were sent to Sampled (www.sampled.com; Piscataway, New Jersey) for genotyping, where they were subjected to further quality control tests. Complete details of quality control, imputation, and empirical ancestry assignment are provided in the S1.

### Construction of polygenic scores (PGS) in alcohol‐related traits

To derive PGS in the GAP sample, we obtained summary statistics from statistically well‐powered GWAS of the following alcohol‐related outcomes: AUD (Kranzler et al., 2019), Alcohol Use Disorders Identification Test—Consumption (AUDIT‐C) (Kranzler et al., 2019), drinks per week (DPW) (Saunders et al., 2022), maximum habitual alcohol consumption (MaxAlc) (Deak et al., 2022), and problematic alcohol use (PAU) (Zhou et al., 2023). Details on each of the original GWAS are provided in the Supplementary Material.

As detailed in the Supplementary Material, GAP only had sufficient sample size for analysis with participants empirically assigned to African (AFR), admixed American (AMR), and European (EUR) ancestry. Therefore, we selected any summary statistics available for these ancestry groups. For PAU, we obtained cross‐ancestry PAU summary statistics; EUR‐only statistics were available, but we chose the mixed ancestry results as they were derived from the greatest sample size and were majority (>80%) European. We downloaded AFR and EUR summary statistics for MaxAlc. All ancestry‐specific summary statistics were available for AUD, AUDIT‐C, and DPW: AMR (Hispanic/Latino American), AFR, and EUR. Details on SNP selection and QC steps are provided in the Supplementary Material. We used PRS‐CSx (Ruan et al., 2022) to adjust summary statistic effect sizes and constructed the PGSs using PLINK 1.9 or PLINK 2.0 (Chang et al., 2015) (details in the Supplementary Material).

### Comparing alcohol‐related PGS between clinic and online GAP recruitment groups

Using the PGS described previously, we compared the mean PGS between samples recruited at clinics versus online. Our approach to inferring genetic ancestry is described in detail in the Supplementary Material. Briefly, within each GAP ancestry group and excluding relatives ($\hat{\pi }\geq 0.1875$), we regressed all 20 ancestry‐specific PCs and participant batch code out of each PGS. Residual PGSs were then z‐scored to have a mean of zero and standard deviation of one. Mean comparisons of the adjusted PGS were done using a two‐sample *t*‐test assuming unequal variance. Statistical significance was an FDR‐adjusted *α* = 0.05. Adjustment of *p*‐values was done independently within each ancestry group; thus, AFR and EUR *p*‐values were adjusted for five tests, and AMR *p*‐values for six tests. *t*‐tests were done in R 4.4. More details on PGS construction and analysis can be found in the Supplementary Material.

## RESULTS

**FIGURE 1:**
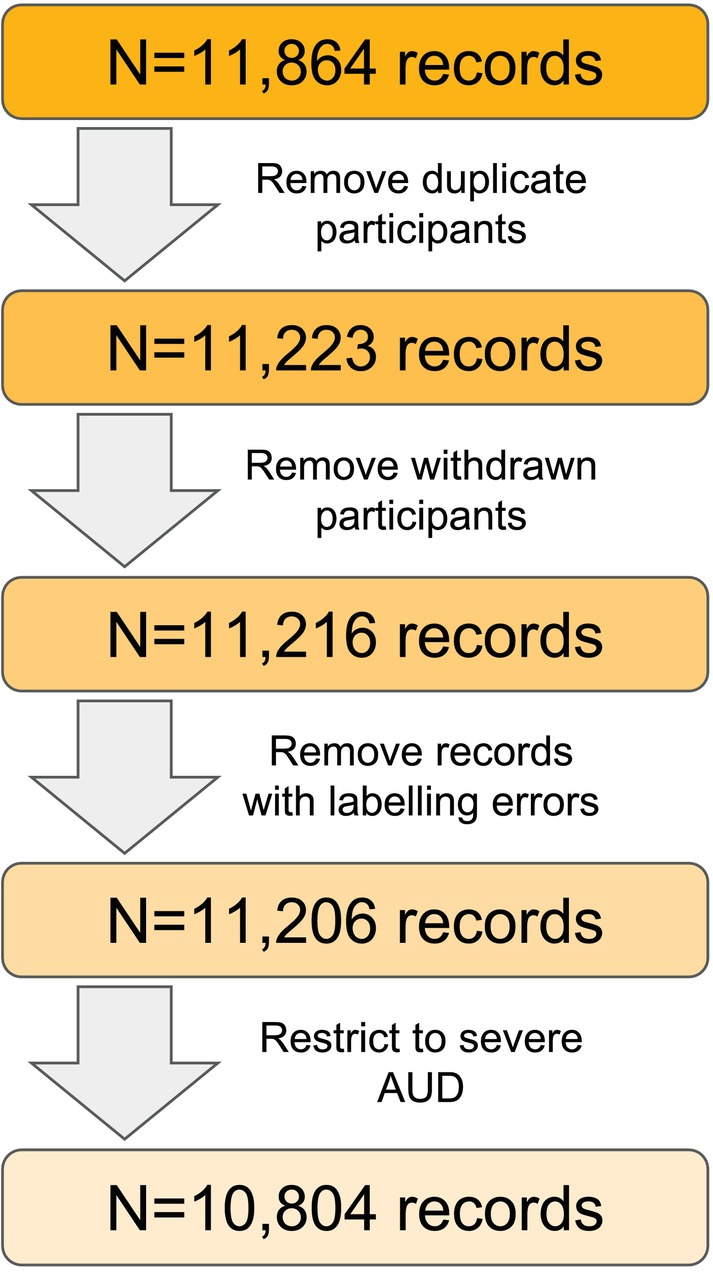
Flowchart of collected records followed by data cleaning for arrival at the total analytic sample size.

We collected a total of *N* = 11,864 records. After removing duplicates (primarily online participants who completed the survey more than once), individuals who wished to withdraw from the study entirely, records with erroneous data, and individuals who did not meet criteria for severe AUD, phenotypic data were available for *N* = 10,804 (Figure 1). Table 1 provides sociodemographic details for the full sample and as a function of ascertainment strategy. The majority of participants (*N* = 9254, 85.7%) were recruited through online sources. Participants from the different ascertainment arms differed in each sociodemographic category, as shown in Table 1. For example, 43% of clinic participants were female, while 66% of online participants were female.

**TABLE 1:** Sample description with sociodemographic data. Data are provided for the total analytic sample and as a function of ascertainment arm.

	Overall sample (*N* = 10,804)		Clinic participants (maximum *N* = 1550)		Online participants (maximum *N* = 9254)		Chi‐squared or *t*‐test statistic and *p*‐value^a^
	*N*	%	*N*	%	*N*	%	
Gender							
Female	6776	0.63	668	0.43	6108	0.66	304.22; *p*_FDR_ = 1.67e‐66
Male	3992	0.37	882	0.57	3110	0.34	
N/A	36		0		36		
Employment							
Full time	4586	0.43	719	0.47	3867	0.42	377.62; *p*_FDR_ = 7.59e‐77
Part time	1321	0.12	122	0.08	1199	0.13	
Student	442	0.04	15	0.01	427	0.05	
Homemaker	449	0.05	52	0.03	507	0.06	
Retired	1145	0.11	69	0.05	1076	0.12	
Disability	1312	0.12	173	0.11	1139	0.12	
Unemployed	1334	0.13	382	0.25	952	0.10	
N/A	105		18		87		
Marital status							
Single	2643	0.25	468	0.31	2175	0.24	66.57; *p*_FDR_ = 5.05e‐12
In a relationship	2057	0.19	213	0.14	1844	0.20	
Married	3822	0.36	592	0.39	3230	0.35	
Divorced/separated	1842	0.17	227	0.15	1615	0.18	
Widowed	325	0.03	32	0.02	293	0.03	
N/A	115		18		97		
Educational attainment							
High school or less	1562	0.15	367	0.24	1195	0.13	143.78; *p*_FDR_ = 2.44e‐29
Some post‐high school	3953	0.37	530	0.35	3423	0.37	
College degree	3014	0.28	418	0.27	2596	0.28	
Graduate degree	2166	0.20	216	0.14	1950	0.21	
N/A	109		19		90		
Race							
AI/AN	140	0.01	15	0.01	125	0.01	120.6; *p*_FDR_ = 9.83e‐23
Asian	204	0.02	9	<0.01	195	0.02	
Black/AA	750	0.07	203	0.13	547	0.06	
NH/PI	23	<0.01	2	<0.01	21	<0.01	
White	9332	0.87	1279	0.83	8053	0.87	
Other	325	0.03	42	0.03	283	0.03	
N/A	30		0		30		
Ethnicity							
Hispanic	654	0.06	53	0.03	601	0.07	22.44; *p*_FDR_ = 9.11e‐05
Non‐Hispanic	10,029	0.94	1496	0.97	8533	0.93	
N/A	121		1		120		
Mean (SD) age	44.93 (13.70)		42.08 (11.74)		45.42 (13.95)		*t* = −10.02; *p*_FDR_ = 1.46e‐21

^a^ Benjamini–Hochberg adjusted false discovery rate *p*‐value.

### Phenotypic analyses

#### Alcohol use behavior

Participants endorsed, on average, 9.10 (SD = 1.77) AUD symptoms, and the mean maximum number of drinks consumed in a 24‐h period was 20.11 (10.13). The mean (SD) age at which participants reported first experiencing problems with alcohol was 25.56 (10.11), with their heaviest period of drinking occurring approximately 5 years later (30.35 [11.27]). The mean score for the Self‐Rating of the Effects of Alcohol for the first five times drinking was 5.18 (2.44) drinks. Participants who had consumed alcohol within the past year had AUDIT‐C scores of 5.84 (3.33) (NB: The possible range of AUDIT‐C scores is 0–40; a score of 8–14 is considered harmful/hazardous, with 15+ indicating AUD). Among those responding to a question regarding their self‐perception of having had alcohol problems, *N* = 796 (7.7%) did not believe they had a problem. Of participants who acknowledged having a problem and reporting its duration (*N* = 10,309), 73.0% indicated that the problem persisted for 1 year or more.

**FIGURE 2:**
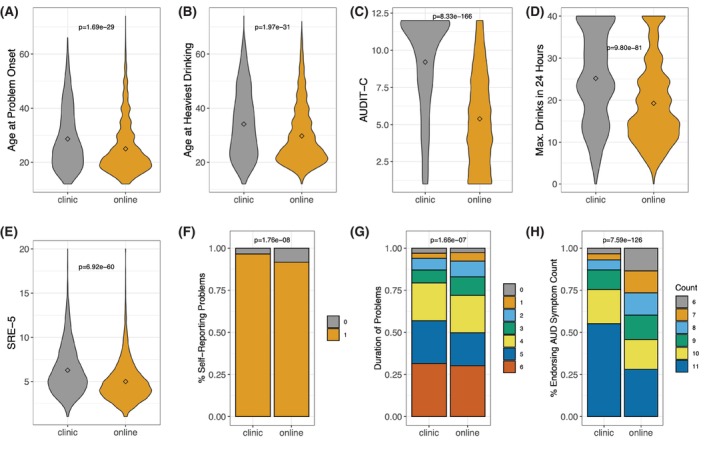
Means (represented by diamonds) and distributions of alcohol‐related variables as a function of ascertainment arm. For continuous variables, *p*‐values from *t*‐tests are presented; for ordinal and categorical variables, *p*‐values from Chi‐square tests are presented; for AUD symptom count, the *p*‐value is from a Wilcoxon signed‐rank test. All *p*‐values are corrected for multiple testing using a Benjamini–Hochberg false discovery rate. Panels are as follows: (A) mean age at onset of alcohol problems; (B) mean age at heaviest drinking; (C) mean AUDIT‐C score; (D) mean maximum number of drinks consumed in 24 h; (E) mean score on the SRE‐5; (F) distribution of participants reporting that they believed they had an alcohol problem; (G) distribution of duration of alcohol problems; (H) distribution of symptom count endorsement. For Figure 2F, 0 = “No” and 1 = “Yes”. For Figure 2G, 0 = “Less than 1 month”; 1 = “1–2 months”; 2 = “3–5 months”; 3 = “6–11 months”; 4 = “1–2 years”; 5 = “3–5 years”; and 6 = “More than 5 years”.

Figure 2A–H depicts distributions of each of these measures as a function of ascertainment strategy. The means or distributions for every outcome varied by ascertainment arm, with clinic participants generally having poorer outcomes (e.g., higher symptom count and maximum number of drinks in 24 h). Clinic participants reported an older onset for problems and period of heaviest drinking.

#### Major depression

**FIGURE 3:**
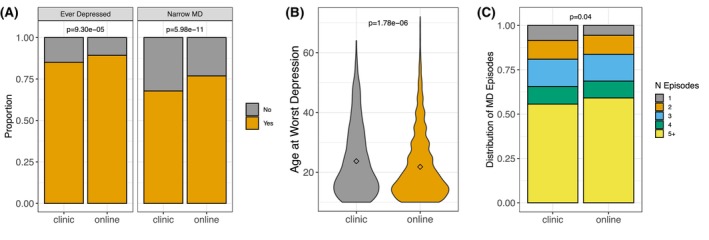
Depression outcomes as a function of ascertainment strategy. For binary or ordinal variables, Chi‐square test *p*‐values are presented; for the continuous outcome, a *t*‐test *p*‐value is reported. All *p*‐values are corrected for multiple testing using a Benjamini–Hochberg false discovery rate. Panel A depicts the distribution of participants who reported ever having felt depressed, and, among those, the proportion meeting a narrow definition of major depression. In panel B, the mean age (represented by a diamond) at the worst depressive episode is depicted. Panel C depicts the distribution of the number of depressive episodes.

Among the full sample, 88.7% reported that they had at some point experienced a period of feeling depressed, down, or sad for at least 2 weeks, with a mean age at worst episode of 22.07 (11.04). Of these respondents, 75.6% met the narrow criteria for MD. The majority of respondents (*N* = 5583, 58.6%) indicated that they had experienced five or more episodes. Clinic participants were less likely to report having felt depressed than online participants, had an older age at worst episode, and reported fewer episodes (Figure 3A–C).

#### Adult antisocial behavior

Participants in the full sample reported a mean of 3.38 (2.56) symptoms of adult antisocial behavior (range 0–7), and 57.6% were classified as meeting diagnostic criteria. Clinic participants were more severely affected (*t* = 12.16, *p*_FDR_ = 2.79e‐31), with a mean (SD) symptom count of 4.10 (2.53), relative to online participants (3.25 [2.54]).

#### Impulsivity

**FIGURE 4:**
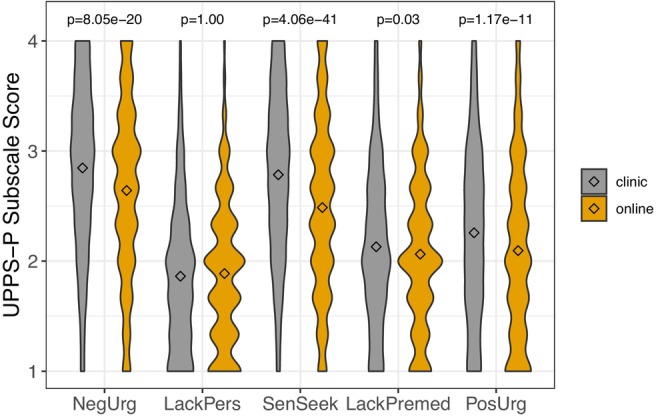
Distributions of scores on the five subscales of the UPPS‐P impulsive behavior scale. The diamonds represent the means for each ascertainment arm. *p*‐Values from *t*‐tests are presented, and were corrected for multiple testing using a Benjamini–Hochberg false discovery rate. NegUrg = negative urgency; LackPers = lack of perseverance; SenSeek = sensation seeking; LackPremed = lack of premeditation; PosUrg = positive urgency.

The possible range for each UPPS‐P subscale was 1–4. For the full sample, the means (SDs) were as follows: negative urgency 2.66 (0.8), lack of perseverance 1.88 (0.65), sensation seeking 2.53 (0.8), lack of premeditation 2.07 (0.7), and positive urgency 2.12 (0.8). As shown in Figure 4, clinic participants exhibited higher scores on each UPPS‐P facet of impulsive behavior, except for lack of perseverance, where there was no difference as a function of ascertainment.

#### Family history of alcohol problems

Overall, 55.0% of the sample reported that their father had an alcohol problem (9.8% reported not knowing), and 31.7% reported that their mother had an alcohol problem (with 5.9% not knowing). A higher proportion of clinic participants reported that their mothers (χ^2^(210696) = 20.76, *p*_FDR_ = 0.001) had alcohol problems, but the difference was not statistically significant for fathers (χ^2^(210701) = 11.15, *p*_FDR_ = 0.16) (Figure S1). Furthermore, when accounting for multiple family members and degree of genetic relatedness, the family history density score was higher among clinic participants (*t* = 4.24, *p*_FDR_ = 9.75e‐04; Figure S2).

#### Other outcomes

Results for outcomes of secondary interest, including smoking and other substance use behavior, personality, and resilience, are provided in the Supplementary Material. Figure S5 provides effect size estimates and confidence intervals for each comparison of phenotypes across ascertainment arms.

### PGS comparison by ascertainment strategy

In the AFR subgroup, the average PGS of AUD, MaxAlc, and PAU was higher among clinic recruits, while for AUDIT‐C and DPW, online participants had a higher mean. However, none of these differences were statistically significant (Table 2, top panel). Among AMR participants, the clinic group had higher average PGS for all phenotypes except DPW (Table 2, middle panel). Again, none of these differences were statistically significant. The MaxAlc phenotype had two PGS derived from AFR + EUR or EUR‐only summary statistics, which had concordant directions of effect. Only in the EUR stratum did PGS exhibit statistically significant differences in mean PGS between recruitment arms (Table 2, bottom panel). The clinic participants had higher mean PGS for AUD (*p*_FDR_ = 8.546 × 10^−10^), AUDIT‐C (*p*_FDR_ = 8.546 × 10^−10^), DPW (*p*_FDR_ = 1.466 × 10^−5^), and PAU (*p*_FDR_ = 0.008). The MaxAlc‐PGS was higher in clinic participants, but these differences were not statistically significant in the EUR group.

**TABLE 2:** Polygenic risk score means for clinic and online recruitment groups in AFR (top panel), AMR (middle panel), or EUR (bottom panel) ancestry.

Phenotype	Ancestry_SS_	Clinic $\bar{x}$(SD)	Online $\bar{x}$(SD)	*N*_Clinic_	*N*_Online_	Df	*t*	*p*_FDR_
AFR ancestry group								
Alcohol use disorder	AFR + EUR	0.047 (0.974)	−0.043 (1.024)	162	178	337.344	0.824	0.811
AUDIT‐C	AFR + EUR	−0.014 (0.967)	0.013 (1.032)	162	178	337.714	−0.242	0.811
Drinks per week	AFR + EUR	−0.014 (1.036)	0.012 (0.969)	162	178	329.427	−0.24	0.811
MaxAlc	AFR + EUR	0.056 (1.005)	−0.051 (0.995)	162	178	334.326	0.982	0.811
Problematic alcohol use	Mixed	0.026 (0.954)	−0.024 (1.042)	162	178	337.984	0.467	0.811
AMR ancestry group								
Alcohol use disorder	AMR + EUR	0.107 (0.893)	−0.019 (1.018)	63	360	92.548	1.007	0.385
AUDIT‐C	AMR + EUR	0.09 (1.153)	−0.016 (0.972)	63	360	78.165	0.686	0.495
Drinks per week	AMR + EUR	−0.201 (0.998)	0.035 (0.998)	63	360	85.145	−1.731	0.244
MaxAlc	AFR + EUR	0.144 (0.845)	−0.025 (1.024)	63	360	96.809	1.419	0.244
MaxAlc	EUR	0.206 (0.888)	−0.036 (1.015)	63	360	92.735	1.952	0.244
Problematic alcohol use	Mixed	0.161 (0.978)	−0.028 (1.003)	63	360	86.398	1.409	0.244
EUR ancestry group								
Alcohol use disorder	EUR	0.182 (1.008)	−0.055 (0.991)	935	3094	1519.584	6.321	8.546 × 10^−10^
AUDIT‐C	EUR	0.179 (0.977)	−0.054 (1.001)	935	3094	1572.198	6.349	8.546 × 10^−10^
Drinks per week	EUR	0.128 (1.008)	−0.039 (0.994)	935	3094	1523.897	4.46	1.466 × 10^−05^
MaxAlc	EUR	0.037 (1.011)	−0.011 (0.997)	935	3094	1523.649	1.296	0.195
Problematic alcohol use	Mixed	0.077 (0.986)	−0.023 (1.003)	935	3094	1563.694	2.725	0.008

Abbreviations: $\bar{x}$, mean; Ancestry_SS_, summary statistic ancestry; AUDIT‐C, alcohol use disorder identification test – consumption; df, degrees‐of‐freedom; MaxAlc, maximum habitual alcohol intake; *p*_FDR_, *p*‐value from *t*‐test after correction for multiple testing using a Benjamini–Hochberg false discovery rate; SD, standard deviation; *t*, *t*‐test statistics.

## DISCUSSION

In the current report, we sought to characterize a large sample of individuals with a lifetime history of severe AUD on a broad range of behavioral phenotypes, and to evaluate differences in those phenotypes as a function of whether participants were ascertained through substance use treatment facilities versus several online avenues. In addition, we compared alcohol‐related polygenic scores across the two ascertainment arms. Participants recruited from clinics versus online differed on nearly all sociodemographic and behavioral outcomes. Clinic‐based participants generally reported more severe adverse outcomes, with notable exceptions, including their lower likelihood of depression. Effect sizes were generally small, suggesting potentially limited clinical utility, although we observed more prominent differences for alcohol‐related measures. In comparisons of genetic liability to alcohol‐related measures, we observed sporadic and inconsistent differences across ascertainment arms, which were significant only among participants of European‐like ancestry. Our findings have implications for participant recruitment in genetic studies of AUD.

Consistent with prior studies, the characteristics of GAP study participants confirms that individuals with severe AUD likely have a history of comorbid depression (Carton et al., 2018; Dawson et al., 2005), other substance use/problems (Grant et al., 2015; Kessler et al., 1997) and antisocial behavior (Grant et al., 2015; Low et al., 2024). In addition, previous research has identified differences in personality/temperament between those with AUD and controls. A meta‐analysis found that individuals with substance use disorders generally score lower on conscientiousness and higher on neuroticism than controls, with no significant differences for extraversion and agreeableness (Kotov et al., 2010). With respect to UPPS‐P facets of impulsivity, positive and negative urgency have been linked to alcohol problems and specific aspects thereof (McCarty et al., 2017; Tran et al., 2018). A meta‐analysis found that positive and negative urgency, along with lack of premeditation, were associated with drinking problems/alcohol dependence, while drinking quantity was associated with lack of perseverance (Coskunpinar et al., 2013). We are unable to corroborate those findings directly in the absence of a control group. Although effect sizes were generally small, it is clear that GAP participants, all of whom have a lifetime history of severe AUD, exhibit variation across the spectrum of psychopathology and related behaviors.

Participants recruited from substance use treatment facilities generally exhibited poorer outcomes relative to online‐recruited participants: Their AUD and antisocial behavior symptom counts were higher, they were more likely to use other substances and to be at high risk for opioid use problems (some participants were recruited from methadone clinics), and they exhibited higher levels of impulsive behavior on several facets. Furthermore, their AUDIT‐C scores (reflecting past‐year consumption) were higher, as was their maximum alcohol intake in a 24‐h period, and they reported a longer duration of problems.

However, there were several exceptions to this trend. Most notably, clinic participants were less likely to report having been depressed or to meet criteria for MD; they also reported fewer depressive episodes than online participants. Relatedly, clinic participants were more likely to agree “a little” or “strongly” that they were able to adapt to change. Less severe issues with depression and higher resilience may reduce likelihood of relapse (Boschloo et al., 2012; Stillman & Sutcliff, 2020; Yamashita et al., 2021). One potential explanation is that the online sample had a higher proportion of females (66% vs. 43%), and females were more likely to meet criteria for MD (79.0% vs. 68.3%). However, a post hoc test indicated that the effect of ascertainment persisted after controlling for sex. We considered alternative explanations, such as the benefits of coping skills or access to behavioral or pharmacotherapy in treatment facilities, but these would be unlikely to explain a difference in lifetime history of MD. Additional research will be necessary to clarify these unexpected findings.

Personality represented another departure from the general observation of poorer outcomes among clinic participants. A previous study reported that individuals with more severe AUD scored higher on neuroticism and extraversion, and lower on agreeableness and conscientiousness (Nilakh et al., 2024). Similarly, a case–control study of AUD found that cases scored higher on neuroticism and lower on conscientiousness (Dash et al., 2019). In the current study, there were no significant differences in neuroticism, but clinic participants scored higher on extraversion, agreeableness, and conscientiousness, which could be interpreted as inconsistent with the notion that clinic participants are more severely affected, or could reflect differences between individuals who are versus are not help‐seeking. However, clinic participants did exhibit higher levels of impulsivity on four of the five UPPS‐P subscales. Profiles of personality and impulsivity are potentially relevant to substance‐related intervention. A recent review found evidence of support for bidirectional relationships between substance use and personality/impulsivity (i.e., one's personality could impact their substance use problems and vice versa), and suggested that neuroticism, conscientiousness, and impulsivity in particular could be effective targets (Juchem et al., 2024). Indeed, such an intervention effort has been applied with some success in a sample of high‐risk adolescents (Newton et al., 2022). The current findings should not be over‐interpreted given small effect sizes, though in conjunction with the prior studies, additional research in this area is warranted.

Our comparisons of PGS across ascertainment arms yielded somewhat inconsistent findings. We observed significant differences by ascertainment arm only among EUR‐like participants. Of five phenotypes tested, one (MaxAlc) did not differ while the remaining (AUD, AUDIT‐C, DPW, and PAU) showed higher PGS among clinic participants. AUD and AUDIT‐C summary statistics are derived from the Million Veterans Program (MVP). These GWAS are well‐powered but are predominantly male (as is the GAP clinic subsample) and have been subject to both self‐selection and selection by the military; this could lead to challenges interpreting the representativeness of downstream results. Overall, taken at face value, the PGS analyses indicate that clinic participants have a higher genetic liability to alcohol problems, with inconsistent results for alcohol consumption. Yet the presence of null results, even for MaxAlc whose EUR discovery sample was comparable to that of AUD and AUDIT‐C, indicates that differences in aggregate genetic burden as a function of ascertainment arm are not inevitable. Thus, these preliminary findings suggest that the recruitment of participants from a non‐clinical setting is unlikely to compromise efforts to identify AUD risk variants when those participants are severely affected. However, additional studies comparing genetic liability among GAP participants to that of controls will be necessary to provide insight to the scale of differences observed here.

There were no significant differences between PGS by ascertainment arm for AFR‐ or AMR‐like GAP participants. Alcohol‐related GWAS in samples of AFR‐ or AMR‐like ancestry are typically conducted in smaller samples, which compromises statistical power for variant discovery (Gratten et al., 2014). Furthermore, predictive power is reduced when EUR‐centric PGS are tested in samples of other ancestries (Duncan et al., 2019). Thus, the lack of significant findings in AFR‐ and AMR‐like GAP participants is not surprising. Where appropriate, we attempted to mitigate these issues through the use of summary statistics derived from multiple ancestry groups (Peterson et al., 2019; Ruan et al., 2022), although we were limited to majority‐EUR summary statistics for PAU. Whether null results in these underrepresented groups are due only to insufficient power versus to a true absence of statistical differences across ascertainment arms is unclear. However, the direction of effect across arms was largely consistent with those observed in the EUR‐like group, suggesting that power is of some concern. Improving the representativeness of participants in genetic analyses is an ongoing challenge in the field for both biological and ethical reasons (Peterson et al., 2019).

Abundant prior research supports the existence of AUD subtypes (Babor et al., 1992; Del Boca & Hesselbrock, 1996; Leggio et al., 2009; Muller et al., 2020). For example, Cloninger and colleagues (Cloninger et al., 1988, 1996) distinguished individuals with Type I versus Type II alcohol problems based in part on age of onset, internalizing problems, and externalizing problems. While conducting latent class or profile analyses within the GAP sample was outside the scope of the current report, the heterogeneity observed in the sample enables such analyses in future studies. Furthermore, in combination with other samples that were broadly phenotypically assessed, and for whom genetic data are available, genetic analysis of AUD subtypes could yield insight to their distinct biological underpinnings.

We did not directly compare participants who did versus did not provide a saliva sample. Very few clinic participants declined to provide a sample, potentially due to the presence of study personnel who had recruited them and led them through the consent process. Among online participants, 61.9% returned a saliva sample, which is consistent with some prior studies (Dykema et al., 2016; Gatny et al., 2013) and considerably higher than others (Bauer et al., 2004; Kozlowski et al., 2002). We previously reported that, among an earlier data freeze of participants recruited through Facebook, saliva sample provision was associated with several demographic and psychosocial factors (Eglovitch et al., 2025). Future work could further elucidate such differences and consider more targeted recruitment options in an effort to obtain saliva samples more consistently across groups.

We note that, although our analyses provide evidence of widespread differences across participants as a function of ascertainment strategy, most of the effect sizes (depicted in Figure S5 as Cohen's *d* and Cramer's *V*) were small. Many of the discrepancies may therefore not be clinically meaningful. However, the more prominent effect sizes were primarily observed for alcohol‐related measures (AUDIT‐C and SRE‐5 scores, and maximum number of drinks in a 24‐h period), which are of greatest interest given that the entire sample was ascertained for severe AUD. These findings bolster our assertion that, even within an otherwise narrowly defined phenotype, considerable phenotypic variation exists. This extends to genetic variation, though PGS for psychiatric outcomes is currently of quite limited clinical utility (Murray et al., 2020).

### Limitations

We note several limitations to the current report. First, online data collection incorporated Facebook ads—which eventually accounted for 55.5% of participants—beginning in March 2020, coinciding with nationwide pandemic disruptions. This could have impacted participant recruitment and eligibility given evidence of shifts in harmful alcohol use during that time (Killgore et al., 2021; Wardell et al., 2020). However, the current report includes samples collected through December 2023, by which point pandemic‐related disruptions had largely ceased.

Second, despite efforts to recruit racially and ethnically diverse participants, our genetic analyses in AFR‐ and AMR‐like ancestry groups were limited to relatively small samples. We are hopeful that, in conjunction with other samples included in consortia‐based efforts, the GAP sample can contribute to meaningful progress toward equitable representation in genetic analyses of alcohol‐related outcomes.

Third, there could be other factors contributing to differences across ascertainment arms that are not captured by the available data. These include individuals' motivation to participate when approached in person versus via the more passive online recruitment effort; this likely also contributed to overall cooperation, as noted above with the very low percentage of eligible clinic participants who declined to provide a DNA sample. There may also be socioeconomic differences beyond educational attainment that influence willingness to participate. Such variables were excluded in an effort to reduce participant burden, but this precludes a more thorough assessment.

In summary, this large sample of individuals with AUD exhibits phenotypic heterogeneity in its sociodemographic characteristics and with respect to behavioral outcomes, including those related directly to alcohol use. In general, participants who were recruited while actively seeking help for substance use exhibited riskier or more adverse psychological and substance use outcomes, with key exceptions. While clinic participants exhibited slightly higher AUD symptom counts than online participants, all individuals included in the current analyses met criteria for a lifetime history of severe AUD, endorsing at least six AUD symptoms. Differences between the ascertainment arms therefore reflect true psychological and behavioral heterogeneity among individuals with severe AUD. Despite these discrepancies, aggregate genetic risk for alcohol‐related phenotypes differed only inconsistently across recruitment modalities. These results underscore the utility and importance of recruiting participants for genetic studies using complementary ascertainment strategies: the inclusion of individuals who were not currently help‐seeking, but who met our stringent inclusion criteria, improved the representativeness of the sample and will bolster statistical power for subsequent genetic analyses, without the potential dilution of genetic signal observed in samples subjected only to minimal phenotyping.

## FUNDING INFORMATION

This study was supported by NIH grants AA026750, AA027522, and AA030611.

## CONFLICT OF INTEREST STATEMENT

The authors declare no conflicts of interest relevant to this study.

## Supporting information


Data S1

## Data Availability

Research data are not shared.
