# Supplementary material for: Participant ascertainment is differentially related to phenotypic characteristics and alcohol‐related genetic liability in a sample with severe alcohol use disorder
Source: Alcohol Clin Exp Res (Hoboken). 2026 Mar 9;50(3):e70276. doi: 10.1111/acer.70276 (PMC12971253; doi:10.1111/acer.70276)
Supplement: Supplementary file 1 — Data S1 [file ACER-50-0-s001.docx]

**Supplementary Materia****l**

**Participant ascertainment is differentially related to phenotypic characteristics and alcohol-related genetic liability in a sample with severe alcohol use disorder**

Alexis C. Edwards^1^, Kristin Passero^1^, Michelle Eglovitch^2^, Kathryn Polak^1^, Anna Beth Parlier-Ahmad^2^, Enkelejda Ngjelina^2^, Mallory Stephenson^3^, Severine Lannoy^1^, Dace Svikis^2,†^, Kenneth Kendler^1,†^

^1^Department of Psychiatry, Virginia Institute for Psychiatric and Behavioral Genetics, Virginia Commonwealth University, Richmond, VA US

^2^Department of Psychology, Virginia Commonwealth University, Richmond, VA US

^3^Department of Cellular, Molecular, and Genetic Medicine, Virginia Commonwealth University, Richmond, VA US

^†^Co-senior authors

Corresponding author: Alexis C. Edwards, Department of Psychiatry, Virginia Institute for Psychiatric and Behavioral Genetics, Virginia Commonwealth University, Box 980126, Richmond, VA 23298-0126, US. E-mail: alexis.edwards@vcuhealth.org. Ph: +1 804-828-8591

**Supplementary Methods**

*Quality control of GAP genotype data*

GAP participants who provided DNA samples were genotyped on the Affymetrix Axiom Precision Medicine Research array in GRCh37 build across 11 batches. We merged the 11 batches whereupon we had 5776 samples genotyped at 902,560 variants. Quality-control (QC) samples (n=59) were dropped, leaving 5717 genotype samples from GAP participants. (Some participants had been genotyped in duplicate and some only had moderate AUD, but these samples were not removed until later in the QC process; there were 5282 unique participants with severe AUD in the genotype data). Prior to further QC, we excluded variants with overlapping positions, variants with impossible heterozygous calls on the Y chromosome, and mitochondrial or pseudo-autosomal XY region variants. Thus 872,524 variants and 5717 were carried forward for standard QC.

We then imposed the following filters: 98% variant call rate, 0.5% minor allele frequency (MAF), Hardy-Weinburg *p*-value 1e-06, and 95% sample call rate. In the remaining samples and SNPs, we calculated per-sample heterozygosity and excluded participants with estimated heterozygosity more than three standard deviations above or below the cohort mean. We calculated identity-by-descent (IBD) to identify relatives. From each group of individuals related at $\hat{\pi}\geq0.45$, we kept the sample with the least genotype missingness, excluding the others from analysis. Genotyped GAP samples were given a unique genotype identification barcode (IID), which contained within it the participant identifier linking that sample back to its phenotypic data. However, a subset of samples had the same participant identifier in their IID despite having $\hat{\pi}<0.1$. We removed these samples, as we were unable to match these participants to their correct phenotype data entry using the participant identifier. We also removed samples missing phenotypic sex. Finally, we excluded any variant who did not have an Affymetrix variant identifier that could be matched to an rsID number. The post-QC GAP genotype data consisted of 475,989 variants and 5046 individuals (4974 unique participants with severe AUD). All QC was done using PLINK 1.9 (Chang et al., 2015, Purcell and Chang).

*Imputation on the TOPMed Imputation Server*

Genotype imputation was done using the TOPMed Imputation Server (Das et al., 2016, Fuchsberger et al., 2015, Taliun et al., 2021). We converted our genotypes from GRCh37 to GRCh38 genome build. We extracted all non-monomorphic, single-nucleotide polymorphisms (SNPs) that passed QC (n=449,957). Using the UCSC Table Browser, we found the GRCh38 positions for most SNPs. For the SNPs whose positions could not be recovered (n=390), we searched the rsID on NCBI dbSNP under the assumption it had been merged into a new rsID and applied the following: (1) if the SNP had been merged with another rsID and we had both SNPs in GAP, keep the SNP retained by GRCh38, (2) if the SNP had been merged with another rsID and the newer rsID was not in GAP, keep and update its name and base position, (3) if the SNP remained in GRCh38 and had not been mapped over by the Table Browser, keep and update its base position, and lastly (4) drop the SNP if it mapped to a nonstandard chromosome name (e.g., fix, patch, alt). We were able to recover 317 of the SNPs with missing positions, resulting in 449,884 SNPs with available GRCh38 locations.

We selected these SNPs and the QC-passing samples (n=5046) and input them into the McCarthy Imputation Preparation and Checking Toolkit for the TOPMed reference panel (<https://www.chg.ox.ac.uk/~wrayner/tools/>). We used the McCarthy Toolkit output to create VCF files and upload them to the TOPMed Imputation Server. GAP genotypes were imputed using the full mixed ancestry TOPMed panel, and the exported results were limited to $R^{2}\geq0.3$. There were 85302202 imputed variants for 5046 samples. We then applied the same QC standards as we did with the genotype chip data (98% variant call rate, 0.5% MAF, Hardy-Weinburg *p*-value 1e-06, and 95% sample call rate). Variants passing QC were filtered to non-genotyped variants (variants not genotyped in GAP) $R^{2}\geq0.8$ with GRCh37 (n=9,810,018) or GRCh38 (n=9,819,089) positions. We continued working with variants mapped to GRCh37 coordinates, as our summary statistics to create polygenic scores (PGS) used this build. The GRCh37 imputed genotypes were merged with the GAP array genotypes (n=475,989) that had passed prior QC. Then variants were restricted to just non-monomorphic SNPs (n=9,704,196) and any overlapping SNPs were dropped in favor of keeping the array genotype (n=9,689,729), creating the final post-QC dataset. All post-imputation QC was done using bcftools 1.15 (Danecek et al., 2021) or PLINK 1.9.

*Genetic ancestry inference using the 1000 Genomes Project/Human Genetic Diversity Project reference*

Genetic ancestry inference was done using a revised version of the Peterson et al. (2017) approach that uses Mahalanobis distance to assign samples to their closest reference superpopulation. We first combined our pre-QC GAP array data (872,524 variants and 5717 samples) with the gnomAD 1000 Genomes Project/Human Genetic Diversity Project (1KG+HGDP) v3.1.2 reference panel (<https://gnomad.broadinstitute.org/downloads#v3-hgdp-1kg>). This callset contains reference samples from seven superpopulations: Africa (AFR), Americas (AMR), Central and South Asia (CSA), East Asia (EAS), Europe (EUR), Middle East (MID), and Oceania (OCE). To merge, GAP positions were flipped to GRCh38. The merge was restricted to autosomal SNPs with identical alleles in GAP and 1KG+HGDP, or alleles that could be resolved by swapping allele order or strand-flipping. The combined GAP+1KG+HGDP callset was filtered to 90% genotype call rate, 0.5% MAF, 90% sample call rate, and Hardy-Weinburg *p*-value 1e-15. We excluded long regions of high linkage disequilibrium (LD) (Anderson et al., 2010); <https://genome.sph.umich.edu/wiki/Regions_of_high_linkage_disequilibrium_(LD)>. The data underwent linkage disequilibrium pruning to $r^{2}=0.1$.

Genetic principal components (PCs) were derived using EIGENSOFT *smartpca* (Patterson et al., 2006, Price et al., 2006) (<https://github.com/DReichLab/EIG>). The unrelated subset of 1KG+HGDP samples (“Variants and unrelated sample PLINK files-.fam” <https://gnomad.broadinstitute.org/downloads#v3-hgdp-1kg-tutorials>) were used as reference samples to compute PCs, and then the remaining related 1KG+HGDP and GAP samples were projected back into the coordinate space. The first round of PCA was implemented using all unrelated 1KG+HGDP as the reference samples, with five iterations of outlier removal along ten PCs. With each successive iteration, *smartpca* dropped samples from the reference set if their value along any PC exceeded six standard deviations (<https://github.com/DReichLab/EIG/blob/master/POPGEN/README>). After obtaining a list of outliers, these samples were dropped from the reference panel and PCA was re-estimated. The unrelated subset of 1KG+HGDP samples excluding outliers was used as the reference to estimate ten PCs. All other 1KG+HGDP and GAP samples were projected into the coordinate space, ensuring that all available GAP+1KG+HGDP had PCs coordinates to use for ancestry inference.

GAP samples were assigned to their nearest ancestry superpopulation. First, each 1KG+HGDP superpopulation was pruned for within-group outliers based on Mahalanobis distance (MD) to the median calculated using the top four PCs. The MD between individual 1KG+HGDP samples and each superpopulation was calculated; typically, the minimum MD was that of the reference sample’s true superpopulation, with 1.2% (51/3928) having a minimum MD to another superpopulation (**Supplementary Table 4**). Exclusion from the reference group was determined by the MD between each 1KG+HGDP sample and its known superpopulation. If MD exceeded the 99^th^ percentile chi-square statistic with four degrees of freedom (X^2^=13.2767), the sample was removed from the superpopulation. After finalizing each superpopulation reference set, MD to the median was calculated between each GAP sample and each superpopulation using all ten PCs. GAP samples were assigned to the superpopulation with the minimum MD. Within each newly assigned GAP ancestry group, outliers were identified by standardizing all in-group MDs and removing samples at least four standard deviations from the mean (n_assigned_=5638, n_excluded_=79). We then selected only the samples who had passed genotype QC. The total sample sizes per ancestry group in GAP are presented in the final column of **Supplementary Table 5**. Only the AFR (n=372), AMR (n=425), and EUR (n=4092) groups were considered to have sufficient sample size for further analysis. All other groups had fewer than 100 samples.

After sorting GAP samples into their respective ancestry groups, we estimated within-ancestry PCs for the AFR, AMR, and EUR subsets. First, we extracted a set of SNPs LD-pruned to r^2^ < 0.1 for each ancestry group. We also calculated IBD to identify relatives ($\hat{\pi}\geq0.1875$) within each group. Relatives were only found in the AFR and EUR groups. For each pair of AFR relatives, we excluded the sample with more missing data. The EUR subset had larger groups of relatives (i.e., samples related to more than one person), so we excluded all relatives for simplicity. Following a similar process as above, PCA was done using nonrelated samples and five iterations of outlier removal. Outlier samples were excluded, and PCA was repeated with no outlier iterations, outputting the top 20 PCs. The coordinates for related samples and the removed outliers were obtained by projecting those samples back into the PC space. Estimation of within-ancestry PCs was also done using *smartpca*.

*Alcohol-related GWAS summary statistics*

*Drinks per week (DPW).* Summary statistics were obtained from the 2022 meta-genome-wide association study (metaGWAS) done by the GWAS and Sequencing Consortium of Alcohol and Nicotine use (GSCAN) (Saunders et al., 2022). While overall 60 cohorts with more than 3 million participants were included, we used only the results from the DPW analysis (n=2965643). DPW describes the average weekly number of alcoholic drinks across all alcohol types. Summary statistics excluding the 23andMe cohort were available for African (n=8078), American (n=5162), and European ancestry (n=666978; Saunders et al, 2022).

Alcohol use disorder (AUD) and alcohol use disorder identification test – consumption (AUDIT-C). GWAS summary statistics for AUD and AUDIT-C were obtained from the same study performed in the Million Veterans Program (MVP) cohort (Kranzler et al., 2019). AUDIT-C score and AUD diagnoses were obtained from the U.S. Department of Veterans Affairs Electronic Health Records (EHR). The study used an age-adjusted mean AUDIT-C measure which downweighed or upweighted individuals under and over 50 years, respectively. GWAS were independently performed in each major ancestry group available in MVP: African American (n_AUD_=56648, n_AUDIT-C_=56495), Hispanic/Latino American (n_AUD_=14175, n_AUDIT-C_=14112), East Asian American (n_AUD_=164, n_AUDIT-C_=1366), European American (n_AUD_=202004, n_AUDIT-C_=200680), and South Asian American (n_AUD_=190, n_AUDIT-C_=189).

Maximum habitual alcohol consumption (MaxAlc). Summary statistics were obtained from a GWAS in the MVP (Deak et al., 2022). Participants of African- and European-descent were selected for ancestry-specific GWASs. MaxAlc was a self-reported response to the survey question *“In a typical month, what is/was the largest number of drinks of alcohol you may have had in one day?”*. There were 29,132 participants of African ancestry and 218,623 participants of European ancestry.

Problematic alcohol use (PAU). “Problematic alcohol use” is a composite phenotype of AUD and Alcohol Use Disorders Identification Test- Problem (AUDIT-P), justified by the two traits’ high genetic correlation (Zhou et al., 2023). PAU summary statistics were obtained via cross-ancestry meta-analysis in cohorts with either AUD or (AUDIT-P) measures. This study included 1079947 individuals of African (n=122571), Hispanic (n=38962), East (n=13551) and South Asian (n=1716), and European (n=903147 descent. The meta-analysis utilized genotype data from the MVP, UK Biobank, iPSYCH, QIMR Berghofer Medical Research Institute, Psychiatric Genomics Consortium (PGC), and Yale-Penn 3, as well as summary statistics from prior studies with FinnGen and five East Asian cohorts. We used the publicly available trans-ancestry meta-analysis summary statistics.

*SNP selection, effects size adjustment with PRS-CSx, and PGS derivation*

The post-QC GAP genotype data contained only non-monomorphic SNPs that had passed previous variant QC filters and could be mapped to GRCh37 coordinates. There were 9,689,729 SNPs, either array genotyped or imputed, in the final callset. We then filtered to keep only SNPs with rsIDs (which some novel imputed variants did not have) and if any two SNPs had the same rsID but different genomic coordinates we dropped one, preferring to keep any SNP genotyped directly. This left 9,688,816 SNPs in the GAP target data to be checked for overlap with SNPs in the DPW, AUD, AUDIT-C, MaxAlc, and PAU summary statistics.

From the GWAS summary statistics, we dropped non-SNPs, duplicates, and SNPs with complimentary alleles. If quality metrics were available, we filtered the summary statistics to keep MAF ≥ 1% and imputed INFO/R^2^ ≥ 0.8. Then, we compared the SNPs in the summary statistics to the 9,688,816 SNPs available in GAP. SNPs were matched by rsID and, if available, chromosome and base position. We confirmed that the shared SNPs’ alleles were identical or could be reconciled by strand-flipping or swapping allele order in PLINK. Final counts of SNPs shared between GAP and summary statistic data, by phenotype and ancestry group, are reported in **Supplementary Table 6**.

We used PRS-CSx (Ruan et al., 2022) to adjust SNP effect sizes for the PGS (Ruan et al, 2022). PRS-CSx uses Bayesian continuous shrinkage priors to adjust effect sizes and jointly shrinks the effects of SNPs that are in linkage disequilibrium (Ruan et al., 2022, Ge et al., 2019). It also allows the user to input summary statistics from multiple populations to boost PRS predictive ability in non-European target data. For each phenotype, we applied PRS-CSx to the EUR summary statistics, using the EUR 1000 Genomes LD reference panel. [PAU PRS had trans-ancestry summary statistics, but were majority European and thus used the EUR LD reference panel in PRS-CSx]. If non-European summary statistics were available, we also applied PRS-CSx jointly to the AFR or AMR results alongside EUR results using all requisite 1000 Genomes LD reference panels (AFR+ EUR and AMR+EUR). The sample sizes input into PRS-CSx are reported in the above sections that describe each PRS phenotype. For PRS-CSx runs involving DPW AFR+EUR and AMR+EUR summary statistics, we set the phi parameter to 1e-2 due to the small sample sizes of the non-European cohorts; all other runs used the default approach Bayesian approach of learning the phi parameter. We used the meta-analyzed effects sizes output by PRS-CSx for the AFR+EUR and AMR+EUR results. Final counts of SNPs included in the PRS are shown in **Supplementary Table 7**. After adjusted effect sizes were obtained, we used PLINK 1.9 or PLINK 2.0 to create weighted sum scores of participants’ genetic liabilities in the samples who had passed our QC.

*Comparing alcohol-related PRS between clinic and online GAP recruitment groups*

We evaluated whether there were significant differences between the two ascertainment strategies (clinic versus online) with regards to their genetic liability for alcohol-related outcomes. Analyses were restricted to unrelated participants (exclude $\hat{\pi}\geq0.1875$) with (1) inferred AFR, AMR, or EUR genetic ancestry, (2) who passed the external genotyping QC (**Supplementary Table 3**), (3) had matching phenotypic and genetic sex, and (4) had severe AUD (n_AFR_=340, n_AMR_=423, n_EUR=_4029). Tests were run independently in each ancestral stratum.

PGS using each set of adjusted effect sizes (AFR+EUR, AMR+EUR, EUR-only, or mixed ancestry) available for each phenotype were derived in all GAP participants regardless of ancestry. However, in the comparisons presented here, we run within-ancestry analyses utilizing only PGS derived from the most similar ancestral summary statistics. All analyses run in the EUR-ancestry participants used EUR-only (AUD, AUDIT-C, DPW, MaxAlc) or mixed ancestry (PAU) PGS. In our AFR ancestry group, the various PRS were compared using PGSs derived from either the AFR+EUR (AUD, AUDIT-C, DPW, MaxAlc) or mixed ancestry (PAU) summary statistics. The only phenotypes with PGS derived with AMR summary statistics were AUD, AUDIT-C, and DPW. Since AMR is an admixed population, we chose to analyze all other PGS regardless of ancestry when an AMR+EUR PGS was not available. Therefore, in the AMR stratum we compared PGS derived from AMR+EUR (AUD, AUDIT-C, DPW), AFR+EUR (MaxAlc), EUR-only (MaxAlc), and mixed ancestry (PAU) summary statistics.

**Supplementary Results**

Smoking behavior. The majority (N=8418, 78.0%) of the sample reported ever having smoked, with 63.7% having smoked at least 100 cigarettes. Among those who had smoked, the mean FTND score was 5.52 (2.77). The distribution of smoking levels (never smoked, smoked <100 cigarettes, smoked 100+ cigarettes) differed across ascertainment arms (χ^2^(2, 10,798) = 53.73, p_FDR_=9.04e-11), with clinic participants more likely to smoke. However, clinic participants had lower FTND scores than online participants (5.13 [2.57] versus 5.60 [2.80], respectively; t= -5.42, p_FDR_=2.90e-06).

Opioid use. Among individuals who reported ever having used opioids in the overall sample, 23.7% were categorized as being at low risk of opioid involvement (operationalized as a score of 0-3 on the NIDA-Modified Assist V2.0); 48.2% were at moderate risk, and 28.0% were at high risk. These proportions were significantly different across ascertainment arms (χ^2^(2, 10,774) = 181.06, p_FDR_=2.02e-38): 7.4% of clinic and 27.1% of online participants were at low risk, while 37.7% of clinic and 26.0% of online participants were at high risk (**Supplementary** **Figure 3**).

Other illicit substance use. As shown in **Supplementary Table 8**, the majority of participants had illicitly used cocaine, marijuana, sedatives, or stimulants at least once; only hallucinogens had not been tried by a slim majority of the sample, and marijuana was the most commonly used substance. Clinic participants were more likely to have used each type of substance than online participants.

Personality. **Supplementary Table 9** reports the mean scores on each of the four facets of personality assessed for the full sample, as well as stratified by ascertainment arm. Clinic participants had higher scores on extraversion and agreeableness, but conscientiousness and neuroticism did not differ as a function of ascertainment.

Resilience. Responses to items addressing resilience are provided in **Supplementary Figure 4**. In the full sample, 77.0% of participants agreed “a little” or “strongly” that they were able to adapt to change, with a corresponding total of 77.3% indicating that they “tend to bounce back” after facing adversity. Endorsement for “adapt to change” differed as a function of ascertainment arm (χ^2^(3,10,640) = 17.48, p_FDR_=0.02), with a higher proportion of clinic participants indicating they adapt to change, relative to online participants. However, there were no differences across groups for bouncing back (χ^2^(3,10,638) = 4.09, p_FDR_=1.00).

**References**

AMERICAN PSYCHIATRIC ASSOCIATION 2013. *Diagnostic and statistical manual of mental disorders: DSM-5,* Washington, D.C., American Psychiatric Association.

ANDERSON, C. A., PETTERSSON, F. H., CLARKE, G. M., CARDON, L. R., MORRIS, A. P. & ZONDERVAN, K. T. 2010. Data quality control in genetic case-control association studies. *Nat Protoc,* 5**,** 1564-73.

BOHN, M. J., BABOR, T. F. & KRANZLER, H. R. 1995. The Alcohol Use Disorders Identification Test (AUDIT): validation of a screening instrument for use in medical settings. *J Stud Alcohol,* 56**,** 423-32.

CHANG, C. C., CHOW, C. C., TELLIER, L. C., VATTIKUTI, S., PURCELL, S. M. & LEE, J. J. 2015. Second-generation PLINK: rising to the challenge of larger and richer datasets. *Gigascience,* 4**,** 7.

CYDERS, M. A., LITTLEFIELD, A. K., COFFEY, S. & KARYADI, K. A. 2014. Examination of a short English version of the UPPS-P Impulsive Behavior Scale. *Addict Behav,* 39**,** 1372-6.

DANECEK, P., BONFIELD, J. K., LIDDLE, J., MARSHALL, J., OHAN, V., POLLARD, M. O., WHITWHAM, A., KEANE, T., MCCARTHY, S. A., DAVIES, R. M. & LI, H. 2021. Twelve years of SAMtools and BCFtools. *Gigascience,* 10.

DAS, S., FORER, L., SCHONHERR, S., SIDORE, C., LOCKE, A. E., KWONG, A., VRIEZE, S. I., CHEW, E. Y., LEVY, S., MCGUE, M., SCHLESSINGER, D., STAMBOLIAN, D., LOH, P. R., IACONO, W. G., SWAROOP, A., SCOTT, L. J., CUCCA, F., KRONENBERG, F., BOEHNKE, M., ABECASIS, G. R. & FUCHSBERGER, C. 2016. Next-generation genotype imputation service and methods. *Nat Genet,* 48**,** 1284-1287.

DEAK, J. D., LEVEY, D. F., WENDT, F. R., ZHOU, H., GALIMBERTI, M., KRANZLER, H. R., GAZIANO, J. M., STEIN, M. B., POLIMANTI, R., GELERNTER, J. & MILLION VETERAN, P. 2022. Genome-Wide Investigation of Maximum Habitual Alcohol Intake in US Veterans in Relation to Alcohol Consumption Traits and Alcohol Use Disorder. *JAMA Netw Open,* 5**,** e2238880.

DICK, D. M., NASIM, A., EDWARDS, A. C., SALVATORE, J. E., CHO, S. B., ADKINS, A., MEYERS, J., YAN, J., COOKE, M., CLIFFORD, J., GOYAL, N., HALBERSTADT, L., AILSTOCK, K., NEALE, Z., OPALESKY, J., HANCOCK, L., DONOVAN, K. K., SUN, C., RILEY, B. & KENDLER, K. S. 2014. Spit for Science: launching a longitudinal study of genetic and environmental influences on substance use and emotional health at a large US university. *Front Genet,* 5**,** 47.

FUCHSBERGER, C., ABECASIS, G. R. & HINDS, D. A. 2015. minimac2: faster genotype imputation. *Bioinformatics,* 31**,** 782-4.

GE, T., CHEN, C. Y., NI, Y., FENG, Y. A. & SMOLLER, J. W. 2019. Polygenic prediction via Bayesian regression and continuous shrinkage priors. *Nat Commun,* 10**,** 1776.

HARRIS, P. A., TAYLOR, R., MINOR, B. L., ELLIOTT, V., FERNANDEZ, M., O'NEAL, L., MCLEOD, L., DELACQUA, G., DELACQUA, F., KIRBY, J., DUDA, S. N. & CONSORTIUM, R. E. 2019. The REDCap consortium: Building an international community of software platform partners. *J Biomed Inform,* 95**,** 103208.

HARRIS, P. A., TAYLOR, R., THIELKE, R., PAYNE, J., GONZALEZ, N. & CONDE, J. G. 2009. Research electronic data capture (REDCap)--a metadata-driven methodology and workflow process for providing translational research informatics support. *J Biomed Inform,* 42**,** 377-81.

HEATHERTON, T. F., KOZLOWSKI, L. T., FRECKER, R. C. & FAGERSTROM, K. O. 1991. The Fagerstrom Test for Nicotine Dependence: a revision of the Fagerstrom Tolerance Questionnaire. *Br J Addict,* 86**,** 1119-27.

JOHN, O. P. & SRIVASTAVA, S. 1999. The big-five trait taxonomy: history, measurement, and theoretical perspectives. *In:* PERVIN, L. A. & JOHN, O. P. (eds.) *Handbook of Personality: Theory and Research.* 2nd ed. New York: Guilford Press.

KENDLER, K. S., AGGEN, S. H. & PATRICK, C. J. 2012. A multivariate twin study of the DSM-IV criteria for antisocial personality disorder. *Biol Psychiatry,* 71**,** 247-53.

KOTOV, R., GAMEZ, W., SCHMIDT, F. & WATSON, D. 2010. Linking "big" personality traits to anxiety, depressive, and substance use disorders: a meta-analysis. *Psychol Bull,* 136**,** 768-821.

KRANZLER, H. R., ZHOU, H., KEMBER, R. L., VICKERS SMITH, R., JUSTICE, A. C., DAMRAUER, S., TSAO, P. S., KLARIN, D., BARAS, A., REID, J., OVERTON, J., RADER, D. J., CHENG, Z., TATE, J. P., BECKER, W. C., CONCATO, J., XU, K., POLIMANTI, R., ZHAO, H. & GELERNTER, J. 2019. Genome-wide association study of alcohol consumption and use disorder in 274,424 individuals from multiple populations. *Nat Commun,* 10**,** 1499.

PATTERSON, N., PRICE, A. L. & REICH, D. 2006. Population structure and eigenanalysis. *PLoS Genet,* 2**,** e190.

PETERSON, R. E., EDWARDS, A. C., BACANU, S. A., DICK, D. M., KENDLER, K. S. & WEBB, B. T. 2017. The utility of empirically assigning ancestry groups in cross-population genetic studies of addiction. *Am J Addict,* 26**,** 494-501.

PRICE, A. L., PATTERSON, N. J., PLENGE, R. M., WEINBLATT, M. E., SHADICK, N. A. & REICH, D. 2006. Principal components analysis corrects for stratification in genome-wide association studies. *Nat Genet,* 38**,** 904-9.

PURCELL, S. & CHANG, C. *PLINK 1.9* [Online]. Available: <https://www.cog-genomics.org/plink2> [Accessed].

RUAN, Y., LIN, Y. F., FENG, Y. A., CHEN, C. Y., LAM, M., GUO, Z., STANLEY GLOBAL ASIA, I., HE, L., SAWA, A., MARTIN, A. R., QIN, S., HUANG, H. & GE, T. 2022. Improving polygenic prediction in ancestrally diverse populations. *Nat Genet,* 54**,** 573-580.

SAUNDERS, G. R. B., WANG, X., CHEN, F., JANG, S. K., LIU, M., WANG, C., GAO, S., JIANG, Y., KHUNSRIRAKSAKUL, C., OTTO, J. M., ADDISON, C., AKIYAMA, M., ALBERT, C. M., ALIEV, F., ALONSO, A., ARNETT, D. K., ASHLEY-KOCH, A. E., ASHRANI, A. A., BARNES, K. C., BARR, R. G., BARTZ, T. M., BECKER, D. M., BIELAK, L. F., BENJAMIN, E. J., BIS, J. C., BJORNSDOTTIR, G., BLANGERO, J., BLEECKER, E. R., BOARDMAN, J. D., BOERWINKLE, E., BOOMSMA, D. I., BOORGULA, M. P., BOWDEN, D. W., BRODY, J. A., CADE, B. E., CHASMAN, D. I., CHAVAN, S., CHEN, Y. I., CHEN, Z., CHENG, I., CHO, M. H., CHOQUET, H., COLE, J. W., CORNELIS, M. C., CUCCA, F., CURRAN, J. E., DE ANDRADE, M., DICK, D. M., DOCHERTY, A. R., DUGGIRALA, R., EATON, C. B., EHRINGER, M. A., ESKO, T., FAUL, J. D., FERNANDES SILVA, L., FIORILLO, E., FORNAGE, M., FREEDMAN, B. I., GABRIELSEN, M. E., GARRETT, M. E., GHARIB, S. A., GIEGER, C., GILLESPIE, N., GLAHN, D. C., GORDON, S. D., GU, C. C., GU, D., GUDBJARTSSON, D. F., GUO, X., HAESSLER, J., HALL, M. E., HALLER, T., HARRIS, K. M., HE, J., HERD, P., HEWITT, J. K., HICKIE, I., HIDALGO, B., HOKANSON, J. E., HOPFER, C., HOTTENGA, J., HOU, L., HUANG, H., HUNG, Y. J., HUNTER, D. J., HVEEM, K., HWANG, S. J., HWU, C. M., IACONO, W., IRVIN, M. R., JEE, Y. H., JOHNSON, E. O., JOO, Y. Y., JORGENSON, E., JUSTICE, A. E., KAMATANI, Y., KAPLAN, R. C., KAPRIO, J., KARDIA, S. L. R., KELLER, M. C., et al. 2022. Genetic diversity fuels gene discovery for tobacco and alcohol use. *Nature,* 612**,** 720-724.

SCHUCKIT, M. A., SMITH, T. L. & TIPP, J. E. 1997. The Self-Rating of the Effects of alcohol (SRE) form as a retrospective measure of the risk for alcoholism. *Addiction,* 92**,** 979-88.

SHEEHAN, D. V., LECRUBIER, Y., SHEEHAN, K. H., AMORIM, P., JANAVS, J., WEILLER, E., HERGUETA, T., BAKER, R. & DUNBAR, G. C. 1998. The Mini-International Neuropsychiatric Interview (M.I.N.I.): the development and validation of a structured diagnostic psychiatric interview for DSM-IV and ICD-10. *J Clin Psychiatry,* 59 Suppl 20**,** 22-33;quiz 34-57.

TALIUN, D., HARRIS, D. N., KESSLER, M. D., CARLSON, J., SZPIECH, Z. A., TORRES, R., TALIUN, S. A. G., CORVELO, A., GOGARTEN, S. M., KANG, H. M., PITSILLIDES, A. N., LEFAIVE, J., LEE, S. B., TIAN, X., BROWNING, B. L., DAS, S., EMDE, A. K., CLARKE, W. E., LOESCH, D. P., SHETTY, A. C., BLACKWELL, T. W., SMITH, A. V., WONG, Q., LIU, X., CONOMOS, M. P., BOBO, D. M., AGUET, F., ALBERT, C., ALONSO, A., ARDLIE, K. G., ARKING, D. E., ASLIBEKYAN, S., AUER, P. L., BARNARD, J., BARR, R. G., BARWICK, L., BECKER, L. C., BEER, R. L., BENJAMIN, E. J., BIELAK, L. F., BLANGERO, J., BOEHNKE, M., BOWDEN, D. W., BRODY, J. A., BURCHARD, E. G., CADE, B. E., CASELLA, J. F., CHALAZAN, B., CHASMAN, D. I., CHEN, Y. I., CHO, M. H., CHOI, S. H., CHUNG, M. K., CLISH, C. B., CORREA, A., CURRAN, J. E., CUSTER, B., DARBAR, D., DAYA, M., DE ANDRADE, M., DEMEO, D. L., DUTCHER, S. K., ELLINOR, P. T., EMERY, L. S., ENG, C., FATKIN, D., FINGERLIN, T., FORER, L., FORNAGE, M., FRANCESCHINI, N., FUCHSBERGER, C., FULLERTON, S. M., GERMER, S., GLADWIN, M. T., GOTTLIEB, D. J., GUO, X., HALL, M. E., HE, J., HEARD-COSTA, N. L., HECKBERT, S. R., IRVIN, M. R., JOHNSEN, J. M., JOHNSON, A. D., KAPLAN, R., KARDIA, S. L. R., KELLY, T., KELLY, S., KENNY, E. E., KIEL, D. P., KLEMMER, R., KONKLE, B. A., KOOPERBERG, C., KOTTGEN, A., LANGE, L. A., LASKY-SU, J., LEVY, D., LIN, X., LIN, K. H., LIU, C., LOOS, R. J. F., et al. 2021. Sequencing of 53,831 diverse genomes from the NHLBI TOPMed Program. *Nature,* 590**,** 290-299.

VAISHNAVI, S., CONNOR, K. & DAVIDSON, J. R. 2007. An abbreviated version of the Connor-Davidson Resilience Scale (CD-RISC), the CD-RISC2: psychometric properties and applications in psychopharmacological trials. *Psychiatry Res,* 152**,** 293-7.

ZHOU, H., KEMBER, R. L., DEAK, J. D., XU, H., TOIKUMO, S., YUAN, K., LIND, P. A., FARAJZADEH, L., WANG, L., HATOUM, A. S., JOHNSON, J., LEE, H., MALLARD, T. T., XU, J., JOHNSTON, K. J. A., JOHNSON, E. C., NIELSEN, T. T., GALIMBERTI, M., DAO, C., LEVEY, D. F., OVERSTREET, C., BYRNE, E. M., GILLESPIE, N. A., GORDON, S., HICKIE, I. B., WHITFIELD, J. B., XU, K., ZHAO, H., HUCKINS, L. M., DAVIS, L. K., SANCHEZ-ROIGE, S., MADDEN, P. A. F., HEATH, A. C., MEDLAND, S. E., MARTIN, N. G., GE, T., SMOLLER, J. W., HOUGAARD, D. M., BORGLUM, A. D., DEMONTIS, D., KRYSTAL, J. H., GAZIANO, J. M., EDENBERG, H. J., AGRAWAL, A., MILLION VETERAN, P., JUSTICE, A. C., STEIN, M. B., KRANZLER, H. R. & GELERNTER, J. 2023. Multi-ancestry study of the genetics of problematic alcohol use in over 1 million individuals. *Nat Med,* 29**,** 3184-3192.

**Supplementary Table 1.** Description of differences across ascertainment arm.

| **Procedures** | **Clinic** | **Online** |
| --- | --- | --- |
| Recruitment | Strategies vary by site and include: use of flyers and posters, presentations given to patients, and approaching patients directly. | Use of targeted advertisements on social media and recovery spaces. Brief information blurbs and a link to the survey are included in the advertisement. |
| Informed Consent (IC) | Participants provide IC at two stages:   - Survey IC: Obtained prior to launching the survey   DNA IC: If eligible, obtained prior to collecting a DNA sample | Participants provide IC after eligibility has been confirmed through the eligibility screening process. Participants consent to both survey and DNA provision. |
| Eligibility | To participate in the survey portion of the study, participants must meet the following criteria:  Inclusion criteria:   - 18 years of age or older - English proficiency - Current or new client at one of the treatment centers where recruitment is conducted   Exclusion criteria:   - No cognitive deficits, delusions, or other issues that would interfere with the consent process or survey participation   Eligibility for DNA sample participation is determined via survey responses and includes meeting criteria for current or lifetime severe AUD (6 of 11 DSM-5 diagnostic criteria) | To participate in the survey and DNA component of the study, participants must meet the following criteria:  Inclusion criteria:   - 18 years of age or older - English proficiency - Criteria for current or lifetime severe AUD (6 of 11 DSM-5 diagnostic criteria) - Willingness to provide saliva sample for DNA |
| Survey | Surveys are conducted on-site using Computerized Intervention Authoring Software (CIAS) on an iPad or tablet. An expressive narrator (avatar) guides participants through the survey | Surveys are completed online using a REDcap (Harris et al., 2019, Harris et al., 2009) link. REDCap (Research Electronic Data Capture) is a secure, web-based software platform designed to support data capture for research studies, providing 1) an intuitive interface for validated data capture; 2) audit trails for tracking data manipulation and export procedures; 3) automated export procedures for seamless data downloads to common statistical packages; and 4) procedures for data integration and interoperability with external sources. |
| Compensation | Participants are compensated with gift cards based on their level of participation:   - $5 gift card for completing the survey   An additional $5 gift card for providing a DNA sample (for a total of $10 in gift cards for participants who complete both parts) | Participants are compensated $10 upon return of the DNA sample. |
| DNA Sample Acquisition | Eligible participants who provide informed consent for DNA collection will have their samples collected on-site, facilitated by study staff. | Participants are sent a DNA kit after REDcap survey completion, and instructed to return DNA kit to receive compensation. |

**Supplementary Table 2.** Description of survey assessments.

| **Primary Construct** | **Secondary Construct** | **Description** | **Reference where relevant** |
| --- | --- | --- | --- |
| Alcohol | Lifetime Alcohol Use Disorder (AUD) | Participants were assessed for lifetime alcohol use disorder using items from the Mini International Neuropsychiatric Interview with the addition of an item addressing craving to enable a DSM-5 diagnosis. Participants were asked to consider the 12-month period during which their drinking was at its worst. As noted in **Supplementary Table 1**, individuals recruited online were administered a screener to evaluate whether they met criteria for severe AUD (6+ criteria); those recruited in clinics could complete the survey regardless but were only eligible for the DNA component if they endorsed 6+ criteria. Only participants with severe AUD are included in the current analyses. | American Psychiatric Association (2013), Sheehan et al. (1998) |
|  | Past 12-month alcohol use | Participants were administered the first three items from the Alcohol Use Disorders Identification Test (AUDIT-C). We did not include the original skip pattern. We tallied a sum score across the three items. | Bohn et al. (1995) |
|  | Sensitivity to alcohol | We used the Self-Rating of the Effects of Alcohol (SRE-5) to assess participant’s initial sensitivity to alcohol. Responses were winsorized at 20+ drinks. The SRE-5 asks participants to consider the first five times they drank, and report how many drinks were necessary to: (i) feel any effect, (ii) become dizzy or slur their speech, (iii) stumble, or (iv) pass out. Respondents can indicate that a particular experience did not happen in those first times drinking. The number of drinks reported for each of these four items is summed, then divided by the number of items for which a response was provided. | Schuckit et al. (1997) |
|  | Maximum drinks in 24 hours | Participants were asked the highest number of drinks consumed in one 24-hour period, with responses winsorized at 40+ drinks. |  |
|  | Age at onset of problems | Participants were asked to report the age at which their problems began. Responses under age 12 were coded as missing. |  |
|  | Duration of problems | Participants were asked to report the duration of alcohol problems, with options:  0 = Less than one month  1 = 1-2 months  2 = 3-5 months  3 = 6-11 months  4 = 1-2 years  5 = 3-5 years  6 = More than 5 years  7 = NA; I have never had any alcohol problems |  |
|  | Age at heaviest drinking | Participants were asked to report the age at which their drinking was at its heaviest. Responses under age 12 were coded as missing. |  |
|  |  |  |  |
| Demographics | Gender | Participants were asked whether they were male or female. |  |
|  | Race | Participants were asked to self-report their race, with response options based on the US Census: American Indian or Alaskan Native, Asian, Black or African American, Native Hawaiian or Other Pacific Islander, White or Caucasian, or Other |  |
|  | Ethnicity | Participants were asked to self-report their ethnicity: Hispanic or Non-Hispanic |  |
|  | Age | Participants were asked to self-report their age in years |  |
|  | Marital status | Options were: Single, In a relationship, Married, or Divorced/separated |  |
|  | Living arrangement | Options were: With my children and significant other/spouse, With my significant other/spouse only, With my children only, With other family, With friends, Alone, I move around a lot or am homeless, or I live in a group home or assisted living facility |  |
|  | Employment | Options were: Full time, 40 hours/week; Part time; Not working due to medical or mental health disability; Retired; Unemployed; Student; Homemaker or stay-at-home mom |  |
|  |  |  |  |
| Smoking behavior | Lifetime smoking behavior | 1 = I have smoked 100+ cigarettes  2 = I have smoked <100 cigarettes  3 = I have never smoked cigarettes |  |
|  | Age at onset | Age at which the participant first smoked a whole cigarette: 1 = 8 or younger  2 = 9-10  3 = 11-12  4 = 13-14  5 = 15-16  6 = 17 or older |  |
|  | Current smoker | 0 = No  1 = Yes |  |
|  | Nicotine dependence | Participants were asked to complete the 6 items in the Fagerström Test for Nicotine Dependence (FTND) based on their period of heaviest smoking. FTND scores were computed based on existing guidance. | Heatherton et al. (1991) |
| Opioid use | Lifetime use | 0 = Never  1 = Once or twice  2 = Monthly  3 = Weekly  4 = Daily or almost daily |  |
|  | Opioid use disorder risk level | Participants were administered opioid items from the NIDA-Modified ASSIST V2.0, with the modification that they were instructed to consider their period of heaviest use. Scores were summed, per existing guidance, to determine whether they were at lower, moderate, or high risk of an opioid use disorder. Only those who endorsed using opioids at least once or twice were administered the relevant items. | https://nida.nih.gov/sites/default/files/pdf/nmassist.pdf |
| Other illicit substance use | Lifetime use | For marijuana, cocaine, stimulants, sedatives, and hallucinogens, participants were asked how many times in their lives they had used each substance:  0 = Never  1 = 1-5 times  2 = 6 or more times |  |
|  | Weekly use | For each substance above, participants were asked to report how often in a week they used the substance during the period when use was at its heaviest or worst:  0 = Less than 1 day/week  1 = 1 day/week  2 = 2 days/week  3 = 3 days/week  4 = 4 days/week  5 = 5 days/week  6 = 6 days/week  7 = Every day or nearly every day |  |
|  | Daily use | For each substance above, participants were asked to report how often each day they used the substance during the period when use was at its heaviest or worst:  1 = Once per day  2 = 2-3 times/day  3 = 4-5 times/day  4 = 6-7 times/day  5 = 8+ times/day |  |
|  | Primary drug of choice | Participants were asked to identify the illicit drug class that had caused the most problems for them. They were then asked five yes/no questions pertaining to the 12-month period of their heaviest use:   - Did they experience tolerance to that drug? - Did they experience withdrawal symptoms when they stopped using it? - Did they use that drug or another drug to prevent withdrawal symptoms, or to feel better? - Did they spend substantial time (>2 hours) obtaining, using, recovering from, or thinking about the drug? - Did they spend less time working, enjoying hobbies, or being with family or friends because of their use of that drug? |  |
| Caffeine use | Current use of caffeinated drinks | Participants were asked if they drink a beverage with caffeine it is (coffee, tea, cola, energy drinks) every day:  0 = No  1 = Yes |  |
|  | Current weekly coffee use | 0 = I don’t drink coffee  1 = <1 day/week  2 = 1 day/week  3 = 2 days/week  4 = 3 days/week  5 = 4 days/week  6 = 5 days/week  7 = 6 days/week  8 = Every day or nearly every day |  |
|  | Daily cups of coffee | 0-10 = 0-10  11 = More than 10 |  |
|  | Weekly energy drink use | 0 = I don’t drink caffeine energy drinks or shots  1 = <1 day/week  2 = 1 day/week  3 = 2 days/week  4 = 3 days/week  5 = 4 days/week  6 = 5 days/week  7 = 6 days/week  8 = Every day or nearly every day |  |
|  | Daily energy drink use | 0-10 = 0-10 |  |
|  | Energy drinks and alcohol | Participants were asked whether they ever combined energy drinks with alcohol:  0 = No, never  1 = Yes, but only 1-2 times  2 = Yes, occasionally (less than once per week)  3 = Yes, frequently (at least once per week) |  |
|  |  |  |  |
| Personality | Overview | Items were selected from the Big Five Inventory for neuroticism, extroversion, agreeableness, and conscientiousness. To minimize participant burden and maximize informativeness, these items were reduced based on an item response analysis conducted in a previous study (Dick et al., 2014). Furthermore, openness was not assessed due to prior evidence that it is not meaningfully related to psychopathology (Kotov et al., 2010), including substance use disorders. All items had Likert response options as follows:  1 = Disagree strongly  2 = Disagree a little  3 = Neither agree nor disagree  4 = Agree a little  5 = Agree strongly  Items were reverse coded as necessary, and summed to obtain a score for each construct. | John and Srivastava (1999) |
|  | Extraversion | I see myself as someone who…   - is talkative - tends to be quiet - is outgoing and sociable |  |
|  | Agreeableness | I see myself as someone who…   - is helpful and unselfish with others - is considerate and kind to almost everyone - is sometimes rude to others |  |
|  | Conscientiousness | I see myself as someone who…   - does a thorough job - is a reliable worker - does things efficiently |  |
|  | Neuroticism | I see myself as someone who…   - can be moody - is relaxed, handles stress well - worries a lot - gets nervous easily - is emotionally stable, not easily upset - can be tense |  |
|  |  |  |  |
| Impulsivity | Overview | Participants were administered items from the UPPS-P, which captures four facets of impulsivity: Premeditation (lack of), Urgency, Sensation Seeking, Perseverance (lack of). All items had Likert response options as follows:  1 = Disagree strongly  2 = Disagree a little  3 = Agree a little  4 = Agree strongly  Items were reverse coded as necessary, and summed to obtain a score for each construct. Urgency was further subcategorized into negative and positive. | Cyders et al. (2014) |
|  | Urgency | - When I feel bad I will often do things I later regret in order to make myself feel better now. (negative) - I tend to lose control when I am in a great mood. (positive) - When I am upset I often act without thinking. (negative) - Others are shocked or worried about the things I do when I am feeling very excited. (positive) - When I feel rejected, I will often say things that I later regret. (negative) - I tend to act without thinking when I am really excited. (positive) |  |
|  | Premeditation | - My thinking is usually careful and purposeful. - I like to stop and think things over before I do them. - I usually think carefully before doing anything. |  |
|  | Perseverance | - I generally like to see things through to the end. - Unfinished tasks really bother me. - I finish what I start. |  |
|  | Sensation seeking | - I quite enjoy taking risks. - I welcome new and exciting experiences and sensations, even if they are a little frightening and unconventional. - I would enjoy the sensation of skiing very fast down a high mountain slope. |  |
|  |  |  |  |
| Resilience | Overview | Participants were administered the two items of the abbreviated Conor-Davidson Resilience Scale (CD-RISC2):   - I am able to adapt to change. - I tend to bounce back after illness and hardship.   Items were scored on a Likert scale:  1 = Disagree strongly  2 = Disagree a little  3 = Agree a little  4 = Agree strongly | Vaishnavi et al. (2007) |
|  |  |  |  |
| Adult antisocial behavior | Overview | Participants were administered 17 items about the frequency of behaviors they had exhibited since turning 18, which corresponded to DSM-IV symptoms for antisocial personality disorder. The response options were:  0 = Never  1 = 1-2 times  2 = 3-5 times  3 = More than 5 times  Where participants had engaged in the behavior at least 3 times, they were further asked whether they did so under the influence of drugs or alcohol, with the following response options:  0 = Never  1 = Sometimes under the influence  2 = Always under the influence  The antisocial behavior (ASB) symptom count was calculated by categorizing each item into corresponding DSM-IV symptoms, of which there are 7. If participants skipped all items corresponding to a particular symptom, they were coded as missing for that symptom. The number of symptoms endorsed was summed, and was coded as missing if >50% of symptoms were missing. If data were incomplete but available for at least 50% of symptoms, a pro-rated symptom count was calculated. A pseudo-diagnosis of adult antisocial behavior was calculated, wherein individuals with a pro-rated score of 3 or greater were coded as meeting criteria. | Kendler et al. (2012) |
|  |  |  |  |
|  |  |  |  |
| Depressive symptoms | Lifetime major depression | Lifetime depressive symptoms are measured using 13 items that address the 9 criteria for DSM-5 major depressive disorder (MD). Participants were first asked, “Thinking back over your entire life, have you ever had a time when you were feeling depressed, down, or sad most of the time for at least two weeks?” This first question had two response options:  0 = No, never  1 = Yes  Only participants who responded affirmatively to the initial question were asked the remaining symptom-based questions, which had response options of:  0 = No  1 = Sometimes  2 = Yes, most of the time | American Psychiatric Association (2013) |
|  | Episodes lasting 2+ weeks | Participants were asked how many times they had experienced many of these symptoms for at least two weeks:  1 = 1 time  2 = 2 times  3 = 3 times  4 = 4 times  5 = 5+ times |  |
|  | Duration of depressive symptoms | Participants were asked how long the symptoms lasted during the worst episode:  1 = Less than 2 weeks  2 = 2-4 weeks  3 = 1-6 months  4 = 7-12 months  5 = More than a year  6 = All my life |  |
|  | Interference | Participants were asked whether their feelings interfered with their daily tasks:  0 = No  1 = Sometimes  2 = Yes, most of the time |  |
|  | Age of onset | Participants were asked the age at which their worst period of depression started. Winsorized below age 10. |  |
|  | Depression symptom count | After categorizing each depression item into a symptom (some symptoms are addressed by multiple items), those symptoms are summed. Possible range 0-9. Participants missing data on more than half the symptoms were coded as missing. Others were pro-rated based on the number of responses. |  |
|  | Narrow MD | Determines if participant met the following criteria: sadness/loss of interest endorsed; 5+ total depression symptoms endorsed (based on the pro-rated symptom count); symptoms lasted at least 2 weeks; experienced interference. Scored 0/1. |  |
|  |  |  |  |
| Family history of alcohol problems | Overview | Participants were asked whether, in their opinion, members of their family had ever had a problem with alcohol. Questions were administered separately for parents, grandparents, full siblings, half siblings, offspring, and aunts/uncles. Where appropriate, participants could indicate that they did not have any relatives of that kind (e.g., siblings). Participants could respond yes, no, or not sure. |  |
|  | Family history based genetic risk score | Based on the number of family members available for each participant, the genetic relationship between each family member (e.g., 50% relatedness for parents, full siblings, and children; 25% for grandparents, half-siblings, and avunculars), and the proportion of those family members who potentially had an alcohol problem, we created a family history based genetic risk score. Due to skewness in the data, this score is standardized using the *scale* option in R. |  |

**Supplementary Table 3.** Flow of sample size from full analytic sample to subsample included in genetic analyses.

| **Step** | **Sample Size** | **Notes** |
| --- | --- | --- |
| Full analytic sample | 10,804 |  |
| Saliva sample provided | 7195 |  |
| DNA extraction attempted | 6355 | Some samples were returned in an unusable state; others have not yet been processed as of submission |
| Passed internal pre-genotyping QC | 6183 | Samples can fail due to low volume or low quality |
| Sent for external genotyping | 5505 | Not all samples have been sent for genotyping |
| Passed external genotyping QC | 5283 |  |
| Passed sample missingness | 5271 | Restricted to unique participant identifiers, as some samples were genotyped multiple times |
| Passed sample sex heterogeneity | 5135 |  |
| Passed IBD/duplicate QC | 4974 |  |

**Supplementary Table 4.** Assigned superpopulation based on Mahalanobis distance (rows) and true superpopulation (columns) in 1000 Genomes Project/Human Genetic Diversity Project reference samples. AFR=Africa, AMR=Americas, CSA=Central and South Asia, EAS=East Asia, EUR=European, MID=Middle East, OCE=Oceania

|  | **AFR** | **AMR** | **CSA** | **EAS** | **EUR** | **MID** | **OCE** |
| --- | --- | --- | --- | --- | --- | --- | --- |
| **AFR** | 948 | 0 | 0 | 0 | 0 | 0 | 0 |
| **AMR** | 0 | 520 | 0 | 0 | 51 | 0 | 0 |
| **CSA** | 0 | 0 | 754 | 0 | 0 | 0 | 0 |
| **EAS** | 0 | 0 | 0 | 781 | 0 | 0 | 0 |
| **EUR** | 0 | 0 | 0 | 0 | 690 | 0 | 0 |
| **MID** | 0 | 0 | 0 | 0 | 0 | 156 | 0 |
| **OCE** | 0 | 0 | 0 | 0 | 0 | 0 | 28 |

**Supplementary Table 5.** Inferred genetic ancestry of GAP participants. MD=Mahalanobis distance, SD=standard deviation, AFR=Africa, AMR=Americas, CSA=Central and South Asia, EAS=East Asia, EUR=Europe, MID=Middle East

|  | **Standard deviations from mean MD** | | | | **Group Total** | **Group Total (excl. SD ≥ 4)** |
| --- | --- | --- | --- | --- | --- | --- |
|  | **SD < 2** | **SD [2-3)** | **SD [3-4)** | **SD ≥ 4** |  |  |
| **AFR** | 357 | 13 | 2 | 4 | 376 | 372 |
| **AMR** | 415 | 10 | 0 | 5 | 430 | 425 |
| **CSA** | 64 | 2 | 2 | 0 | 68 | 68 |
| **EAS** | 4 | 0 | 0 | 0 | 4 | 4 |
| **EUR** | 3906 | 108 | 78 | 65 | 4157 | 4092 |
| **MID** | 10 | 0 | 1 | 0 | 11 | 11 |
| **Cumulative Total** | 4756 | 4889 | 4972 | 5046 |  |  |

**Supplementary Table 6.** Number of SNPs shared between GAP and summary statistics by ancestry group. DPW=drinks per week,, AUD=alcohol use disorder, AUDIT-C=alcohol use disorder identification test – consumption, MaxAlc=maximum habitual alcohol intake, PAU=problematic alcohol use, AFR=Africa, AMR=Americas, EUR=Europe

| **Phenotype** |  | **Ancestry group of summary statistics** | | | | | |
| --- | --- | --- | --- | --- | --- | --- | --- |
|  | **AFR-only** | | **AMR-only** | **EUR-only** | **Mixed** | **AFR+EUR** | **AMR+EUR** |
| **DPW** | 5805982 | | 6091965 | 6214899 | -- | 7394901 | 6639135 |
| **AUD** | 2422778 | | 3369843 | 3432553 | -- | 4055722 | 3974741 |
| **AUDIT-C** | 2424197 | | 3426503 | 3433844 | -- | 4056192 | 3982336 |
| **MaxAlc** | 4258707 | | -- | 4550868 | -- | 5902203 | -- |
| **PAU** | -- | | -- | -- | 6917449 | -- | -- |

**Supplementary Table 7.** SNPs retained by PRS-CSx and used to create final polygenic risk scores. DPW=drinks per week, AUD=alcohol use disorder, AUDIT-C=alcohol use disorder identification test – consumption, MaxAlc=maximum habitual alcohol intake, PAU=problematic alcohol use, AFR=Africa, AMR=Americas, EUR=Europe

| **Phenotype** | **Ancestry group of the summary statistics** | | |
| --- | --- | --- | --- |
|  | **EUR-only** | **AFR+EUR** | **AMR+EUR** |
| **DPW** | 1035629 | TBD | TBD |
| **AUD** | 573390 | 618450 | 623504 |
| **AUDIT-C** | 573710 | 618558 | 624566 |
| **MaxAlc** | 604396 | 708518 | -- |
| **PAU** | 1032547 | -- | -- |

**Supplementary Table 8.**  Frequency of illicit drug use, other than opioids, in full analytic sample.

|  | Overall sample  (maximum N=10,804) | | Clinic participants (maximum N=1550) | Online participants  (maximum N=9254) | Chi-square statistic and  P_FDR_-value^1^ |
| --- | --- | --- | --- | --- | --- |
| **Substance** | **Frequency** | **N (%)** | **N (%)** | **N (%)** |  |
| Cocaine | 0 times | 4837 (45%) | 443 (29%) | 4394 (47%) | 266.48, p_FDR_=5.74e-57 |
|  | 1-5 times | 2137 (20%) | 291 (19%) | 1883 (20%) |  |
|  | 6+ times | 3764 (35%) | 811 (52%) | 2953 (32%) |  |
| Hallucinogens | 0 times | 5590 (52%) | 708 (46%) | 4882 (53%) | 33.54, p_FDR_=2.18e-06 |
|  | 1-5 times | 2967 (27%) | 447 (29%) | 2520 (27%) |  |
|  | 6+ times | 2218 (21%) | 391 (25%) | 1827 (20%) |  |
| Marijuana | 0 times | 1113 (10%) | 98 (6%) | 1015 (11%) | 46.78,  p_FDR_=2.92e-09 |
|  | 1-5 times | 2108 (20%) | 260 (17%) | 1848 (20%) |  |
|  | 6+ times | 7557 (70%) | 1190 (77%) | 6367 (69%) |  |
| Sedatives | 0 times | 5230 (48%) | 641 (41%) | 4589 (50%) | 60.27, p_FDR_=3.43e-12 |
|  | 1-5 times | 2341 (22%) | 318 (21%) | 2023 (22%) |  |
|  | 6+ times | 3204 (30%) | 586 (38%) | 2618 (28%) |  |
| Stimulants | 0 times | 5269 (49%) | 660 (43%) | 4609 (50%) | 54.86, p_FDR_=5.12e-11 |
|  | 1-5 times | 2002 (19%) | 256 (17%) | 1746 (19%) |  |
|  | 6+ times | 3502 (32%) | 628 (41%) | 2874 (31%) |  |

^1^Benjamini-Hochberg false discovery rate p-value

**Supplementary Table 9.** Mean (standard deviation) scores for each of the four facets of personality assessed.

| **Facet** | **Overall sample**  **(maximum N=10,804)** | **Clinic participants (maximum N=1550)** | **Online participants**  **(maximum N=9254)** | **t-test statistic and p-value^1^** |
| --- | --- | --- | --- | --- |
| Conscientiousness | 12.45 (2.60) | 12.59 (2.65) | 12.42 (2.59) | 2.34, p_FDR_=0.82 |
| Extraversion | 9.64 (3.47) | 10.36 (3.36) | 9.52 (3.47) | 9.04, p_FDR_=1.41e-17 |
| Agreeableness | 11.60 (2.53) | 12.00 (2.48) | 11.53 (2.53) | 6.90, p_FD_=2.82e-10 |
| Neuroticism | 19.47 (4.43) | 19.57 (4.33) | 19.45 (4.45) | 0.94, p_FDR_=1.00 |

^1^Benjamini-Hochberg false discovery rate p-value

**Supplementary Figure 1.** Endorsement of parental alcohol problems as a function of ascertainment arm.

**Supplementary Figure 2.** Density plot of family history density of alcohol problems. As described in Supplementary Table 1, this score accounts for the number of relatives in a family and for genetic relatedness between the proband and each type of family member. It is standardized for the full analytic sample. Here, the means are compared across ascertainment arms. The p-value, which was corrected for multiple tests using a Benjamini-Hochberg false discovery rate. is from a t-test.

**Supplementary Figure 3.** Distribution of opioid use risk categories as a function of ascertainment arm.

**Supplementary Figure 4.** Response distributions of participants in the full sample and each ascertainment arm to items addressing resilience.

**Supplementary Figure 5.** Effect sizes and 95% confidence intervals for comparisons of phenotypes across ascertainment arms. For continuous phenotypes, we report Cohen’s *d*, while for binary or categorical phenotypes we report Cramer’s V. The figure does not depict the effect size for alcohol use disorder symptom count, which was the only variable for which we used a Wilcoxon test to compare across ascertainment arms. The estimated effect size (95% confidence intervals) was 0.37 (0.34; 0.40).
